# Nuclear‐Mitochondria Crosstalk in Senescent Adipose‐Derived Stem Cells

**DOI:** 10.1002/smmd.70043

**Published:** 2026-07-13

**Authors:** Yixiang Zhang, Yawei Du, Shifeng Ling, Hanqi Wang, Jiahao He, Jiahong Li, Kaifeng Huo, Baikun Liu, Qingfeng Li, Jiao Wei, Wenguo Cui

**Affiliations:** ^1^ Shanghai Key Laboratory for Prevention and Treatment of Bone and Joint Diseases, Department of Orthopaedics Shanghai Institute of Traumatology and Orthopaedics Ruijin Hospital Shanghai Jiao Tong University School of Medicine Shanghai China; ^2^ Department of Plastic & Reconstructive Surgery Shanghai Ninth People's Hospital Shanghai Jiao Tong University School of Medicine Shanghai China; ^3^ Department of Radiology Ruijin Hospital Shanghai Jiao Tong University School of Medicine Shanghai China; ^4^ Department of Infectious Diseases Massachusetts General Hospital Harvard Medical School Broad Institute Boston Massachusetts USA

**Keywords:** adipose derived stem cell, aging, cellular communication, mitochondria, mtDNA

## Abstract

Adipose‐derived stem cells (ADSCs) are central regulators of adipose tissue homeostasis and regenerative capacity. Accumulating evidence indicates that aging and obesity profoundly impair ADSC function, through progressive mitochondrial dysfunction and disrupted mitochondrial–nuclear communication. Emerging studies reveal that defects in nuclear–mitochondrial crosstalk constitute a key driver of ADSC senescence and adipose tissue aging. In this review, we synthesize recent advances in understanding the mitochondrial mechanisms underlying ADSC aging, with particular emphasis on how mitochondrial dysfunction reshapes stem cell fate decisions, metabolic plasticity, and inflammatory signaling within aged adipose niches. We further highlight mitochondria targeting therapeutic strategies that hold promise for reversing ADSC senescence. Collectively, this framework positions mitochondrial regulation as a unifying axis for ADSC rejuvenation, offering new opportunities to restore adipose tissue homeostasis and mitigate age‐related metabolic dysfunction.

## Key Points

- ADSC senescence is driven by mitochondrial dysfunction, leading to impaired adipose tissue homeostasis and regenerative capacity during senescence.
- Bidirectional nuclear–mitochondria crosstalk, including anterograde and retrograde signaling pathways, critically regulates ADSC fate, metabolism, and inflammatory responses in senescence.
- Targeting mito–nuclear communication through mitochondrial regulation and emerging organelle‐ and genome‐engineering strategies offers promising therapeutic avenues for ADSC rejuvenation and age‐related adipose disorders.

## Graphical abstract

Aging and obesity disrupt bidirectional nucleus–mitochondria communication in adipose‐derived stem cells (ADSCs). Enhanced anterograde and retrograde signaling drives mitochondrial dysfunction, nuclear reprogramming, and cellular senescence, ultimately contributing to ADSC senescence and impaired adipose tissue homeostasis.

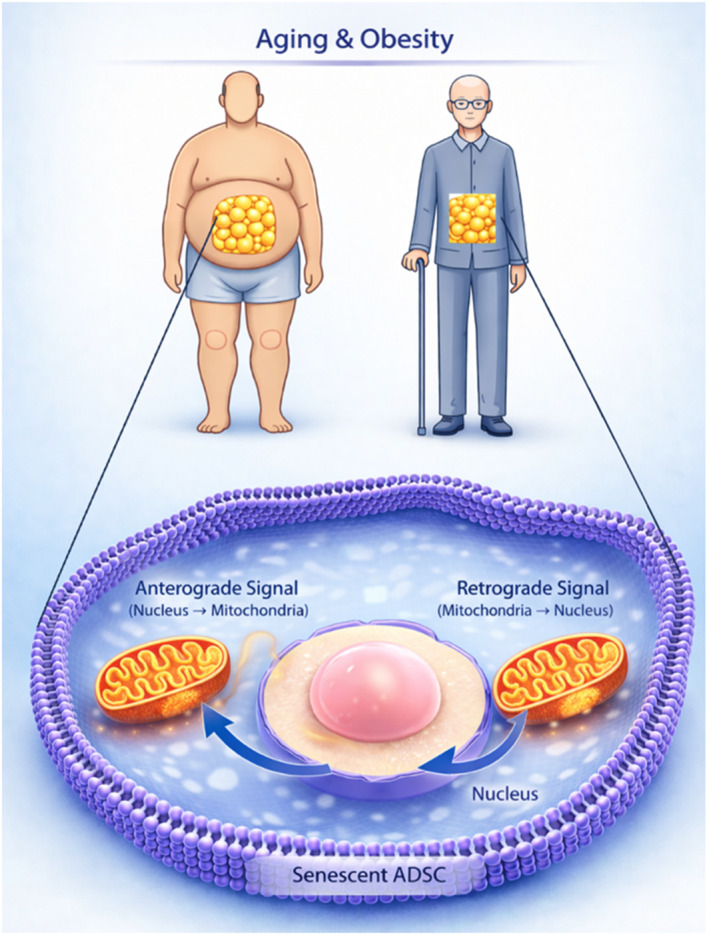

## 1 Introduction

Adipose‐derived stem cells (ADSCs) are a type of multipotent mesenchymal stem cell derived from adipose tissue, with broad potential applications in regenerative medicine and anti‐aging research [1]. As the sole stem cell population within adipose tissue, ADSCs are essential for preserving adipose tissue homeostasis [2]. As the largest endocrine organ in humans, adipose tissue plays a central role in metabolic regulation, and its dysfunction leads to marked metabolic imbalance [3]. However, with aging or the onset of obesity, senescent ADSCs exhibit gradually diminished functionality, including reduced differentiation and proliferation capacity, weakened stress response, and further disruption of adipose tissue homeostasis [2, 4, 5, 6]. Therefore, in‐depth exploration of the regulatory mechanisms of ADSC senescence and potential intervention strategies is of great significance to restore adipose tissue function and maintain metabolic balance in the body.

Mitochondria, as the central organelles for cellular energy metabolism and signaling, are closely linked to cellular senescence [7]. In senescent cells, mitochondrial dysfunction disrupts essential physiological functions and exacerbates the aging process [8]. In recent years, the intricate bidirectional communication between the nuclear and mitochondria is highlighted, known as nuclear‐mitochondria crosstalk, as a key player in regulating cellular homeostasis during senescence [9]. Understanding how this nuclear‐mitochondrial communication deteriorates in senescent ADSCs and identifying potential interventions to restore it could provide novel opportunities for therapeutic approaches aimed at reversing cellular aging and improving adipose tissue function [10].

This review focuses on the latest advances in understanding nuclear‐mitochondrial crosstalk in senescent ADSCs. We begin by summarizing the current knowledge of aging, adipose tissue aging and the role of ADSCs in maintaining tissue homeostasis. We then explored the pathways involved in nuclear‐mitochondria communication and their dysregulation in senescent ADSCs, with a particular focus on two key signaling pathways. Finally, we discuss emerging therapeutic opportunities targeting this crosstalk, aiming to restore ADSC function and promote adipose tissue homeostasis. We present an ADSC‐centered review of bidirectional mito‐nuclear crosstalk in senescent ADSCs, structured by anterograde and retrograde signaling. We further connect these signaling axes to ADSC‐relevant senescence outcomes, including metabolic reprogramming, reduced adipogenic capacity, and senescence‐associated secretory phenotype (SASP)‐associated remodeling. By highlighting these insights, this review seeks to provide a foundation for future research into anti‐aging therapies and regenerative medicine.

## 2 Adipose Aging and ADSC Senescence

### 2.1 Adipose Aging

Aging represents a multifactorial biological process in which physiological integrity gradually deteriorates, ultimately leading to functional impairment and increased risk of disease and death [11]. It is driven by a variety of interconnected cellular and molecular mechanisms, including genome instability, telomeric erosion, epigenetic dysregulation, compromised proteostasis, altered nutrient‐sensing pathways, mitochondrial impairment, cellular senescence, exhaustion of stem cell pools, and aberrant intercellular signaling [12, 13, 14, 15]. These hallmarks collectively contribute to tissue degeneration, chronic inflammation, and a decreased capacity for regeneration [5, 12]. Elucidating the fundamental mechanisms underlying aging is critical not only for delaying age‐related functional decline but also for developing targeted interventions that may promote healthy aging and extend lifespan.

Different organs and tissues age at varying rates [16]. Recent studies have increasingly indicated that adipose tissue responds significantly to aging‐related changes through heterochronic parabiosis and multi‐tissue single‐cell analysis [16, 17, 18, 19, 20]. Firstly, widespread immune cell activation was detected and most pronounced in white adipose tissue [21]. In addition, the stem cell population of adipose tissue represents one of the most sensitive cells to aging environment [21]. These findings suggest that adipose tissue plays a critical role in mediating aging‐related systemic changes and modulating the risk of disease related to aging. Aging‐induced systemic decline may be mediated through multiple mechanisms in adipose tissue [22].

**FIGURE 1:**
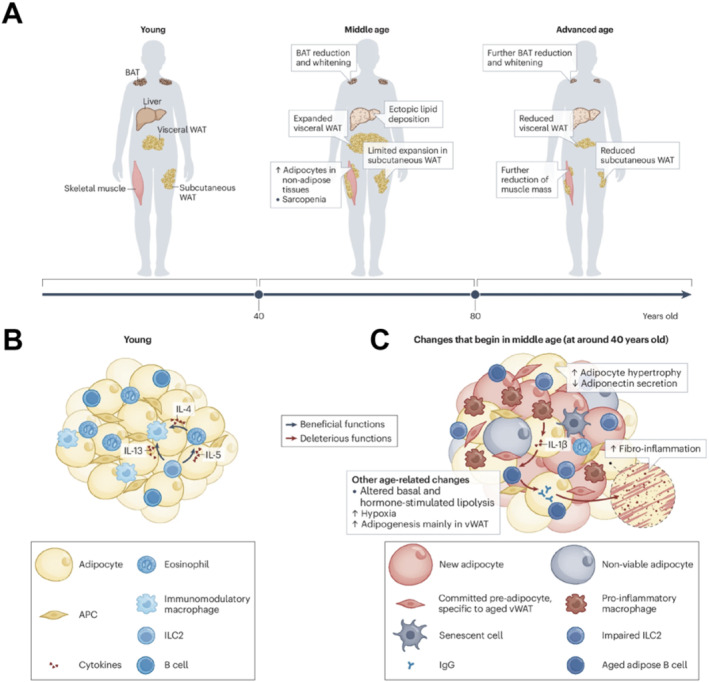
Age‐associated structural, metabolic, and immunological remodeling of adipose tissue. (A) Systemic anatomical and metabolic changes in adipose depots across the lifespan. In young individuals, SAT predominates, BAT is active, and skeletal muscle mass is preserved. Beginning in middle age (∼40 years), BAT volume and activity decline, visceral WAT expands, subcutaneous WAT shows limited plasticity, and ectopic lipid deposition emerges, accompanied by increased adipocytes in non‐adipose tissues and early sarcopenia. In advanced age, BAT further decreases and SAT and VAT progressively shrink. (B) Cellular and immunological landscape of young adipose tissue. Young WAT is characterized by a balanced microenvironment composed of adipocytes, APCs, immunomodulatory macrophages, ILC2s, eosinophils, and B cells. High levels of homeostatic cytokines support anti‐inflammatory signaling, tissue remodeling, and metabolic equilibrium. (C) Age‐related inflammatory remodeling beginning around middle age. Middle‐aged WAT exhibits adipocyte hypertrophy, reduced adiponectin secretion, increased IL‐1β signaling, and fibro‐inflammatory changes. Modified and reprinted with permission [28]. Copyright 2025, Springer Nature Limited.

A number of conserved changes occur during adipose aging. Adipose tissue can be broadly categorized into three principal forms, namely subcutaneous (SAT), visceral (VAT), and brown adipose tissue (BAT). SAT is mainly responsible for the storage and release of triglycerides and fatty acids, and when the function of SAT is impaired, it may lead to elevated levels of lipids in the blood, which in turn accumulate in non‐adipocytes, triggering lipotoxicity and insulin resistance [23, 24, 25]. VAT is located in the peritoneal cavity and is distributed among different sites, including the greater omentum, mesentery, and gonadal regions [26, 27]. BAT is a specialized type of adipose tissue primarily involved in non‐shivering thermogenesis, a process by which heat is generated through the uncoupling of oxidative phosphorylation in mitochondria (Figure 1).

### 2.2 Pathological Changes in Adipose Tissue Aging

There are numerous pathological changes associated with adipose tissue aging. For example, with individual age, there is a gradual decrease in the volume of SAT and a decrease in lipid storage capacity. This process manifests itself differently in men and women as aging is influenced by changes in sex hormones [29, 30, 31]. Subcutaneous adipose loss during aging has been observed in a variety of species, including mice, dogs, cats, and nonhuman primates [32, 33, 34, 35]. Even in species lacking adipocytes. Even in flies, the distribution of fat in the head and abdominal fat bodies diminishes with aging [36, 37]. Diminished SAT lipid storage capacity during aging places greater metabolic stress on other cells, affecting the redox state and energy balance, which in turn may promote cellular senescence. Apart from lipid storage, SAT size and functional integrity regulate the circulating levels of key adipokines, including lipocalin and leptin. Studies have shown that circulating lipocalin correlates with lifespan in humans and also has a direct effect on lifespan in mice [38, 39].

Obesity and aging have similar effects on VAT, as evidenced by the tendency of infiltrating and resident immune cells toward a pro‐inflammatory phenotype [40, 41]. VAT levels increase with the aging process and are a major risk factor for age‐related diseases such as type 2 diabetes, cardiovascular disease, stroke, and metabolic syndrome [42].

In summary, different types of adipose tissue display conserved changes during aging, and their functional decline significantly impacts systemic metabolic homeostasis and healthy lifespan.

Among the diverse cellular components of adipose tissue, adipocytes function as potent endocrine cells that secrete a wide range of bioactive peptides and extracellular vesicles [43]. Through these secretory factors, adipocytes modulate the activity of surrounding tissues and organs, thereby exerting broad effects on aging processes and lifespan regulation. Emerging evidence indicates that distinct adipocyte subtypes possess unique secretory profiles, enabling them to exert specific influences on neighboring adipose‐resident cell populations [44, 45]. Despite occupying the majority of adipose tissue volume due to their large size, adipocytes account for less than half of the total cellular population within adipose tissue [46]. In contrast, immune cells, endothelial cells, and adipose‐derived stem cells collectively comprise more than 50% of adipose tissue cells [46]. The infiltration and accumulation of immune and endothelial cells within adipose tissue are considered major contributors to the age‐associated systemic pro‐inflammatory milieu, which can subsequently impair tissue regeneration and disrupt immune homeostasis [47, 48]. Notably, adipose‐derived stem cells not only replenish adipocytes but also retain multilineage differentiation potential, giving rise to cell types such as osteoblasts and chondroblasts [49]. Consequently, age‐related dysfunction of ADSC undermines adipose tissue homeostasis and contributes to its progressive decline.

Through aging, the main change that occurs in adipocytes is a sustained increase in average size, and the increase in adipose tissue can be achieved through adipocyte hyperplasia (hyperplasia) or enlargement of individual adipocytes (hypertrophy) [50, 51]. The hypertrophic properties of adipocytes are particularly pronounced, and their diameter can increase from < 20–300 μm [52]. Adipocyte hypertrophy from either obesity or aging is positively associated with insulin resistance [53]. For immune cells, immune cell infiltration in senescent or obese adipose tissue is a characteristic change and is more pronounced in VAT [54]. In addition, infiltrating immune cells are converted to a pro‐inflammatory phenotype, affecting tissue homeostasis and metabolism and exacerbating systemic insulin resistance [55, 56]. The transition of immune cells to an inflammatory phenotype has been termed “inflammaging.” The conversion of immune cells to an inflammatory phenotype has been termed “inflammaging” [57]. In addition to adipocytes and immune cells, endothelial cells also decline in function in senescent adipose tissue, but they are less sensitive to senescence [58]. Endothelial cell dysfunction is manifested by decreased levels of the key angiogenic factor VEGF in senescent adipose tissue, decreased vascularity and increased hypoxia [59]. The most important cellular senescence in adipose tissue, ADSC senescence, is discussed in detail in the next section.

### 2.3 ADSC Senescence in Adipose Aging

The senescence of ADSCs plays one of the most critical roles in adipose tissue senescence, as their adipogenic differentiation ability directly determines the formation of new adipocytes within the adipose tissue. However, with aging, a reduction in the proliferative and differentiation capacity of ADSCs may lead to aging‐associated adipocyte hypertrophy [60, 61, 62]. Several studies have shown that the effect of aging on the number of ADSCs varies by species and site of adipose tissue. As an example, aging did not alter the number of adipocyte precursor cells in rat adipose tissue [63]. In contrast, in human studies, some studies have reported that the proliferative capacity of subcutaneous adipose tissue progenitor cells was affected by age, but no significant changes were seen in omental adipose tissue, and some studies have reported that the proliferative capacity of both ADSCs was downregulated [64, 65, 66]. These differences may arise from species or adipose tissue type specificity. Furthermore, even if the proliferative capacity of progenitor cells was unaffected in some studies, aging impairs their differentiation capacity [67]. Aging‐associated progenitor cell dysfunction may be associated with reduced levels of key transcription factors (e.g., C/EBP and PPARγ), and related results have been validated in white adipose tissue from aged rats and non‐human primates [68, 69].

Specifically, several factors are involved in the mechanism of functional down‐regulation of adipose‐derived stem cells on differentiation. In addition to aging‐induced downregulation of the levels of key transcription factors, elevated levels of anti‐adipogenic factors, such as C/EBPβ‐LIP, CHOP, and CUGBP1, which are upregulated to inhibit adipocyte differentiation, contribute to the impaired differentiation capacity in the aging process [70, 71]. microRNAs (e.g., miR‐143) also influence preadipocyte dysfunction by regulating transcription and mRNA translation in the adipogenic pathway. Among them, miR‐143 promotes adipocyte differentiation through the ERK5‐PPARγ pathway, which is closely associated with age‐related differentiation defects [72].

The underlying cause of down‐regulation of senescent ADSC function is cellular senescence. In general, both aging organisms and obesity lead to ADSC senescence. Among them, replicative senescence occurs in ADSCs in aging organisms, whereas oxidative stress senescence occurs in ADSCs in obese organisms [73, 74]. Cellular senescence is generally characterized by several changes including cell cycle arrest, genomic instability, telomere shortening, epigenetic changes, proteostatic imbalance, dysregulation of macroautophagy, and mitochondrial dysfunction [12]. It has been shown that senescent ADSCs isolated from obese individuals have a down‐regulated proliferative capacity and express senescence‐associated markers TP53, IL6, and CCL2. Senescent ADSCs also express SA‐β‐gal, as well as the cycle‐blocking‐related proteins CDKN1A, CDKN2A [75], and release of SASP [76]. Cell cycle alterations allow senescent ADSCs to have a shortened G1 phase and this alteration may affect the differentiation propensity of ADSCs [76]. Meanwhile, the chromatin accessibility of senescent ADSCs was more stable, suggesting that their chromatin structure is more strongly regulated [77]. ADSCs are subjected to a variety of stresses during aging, including accumulation of toxic metabolites, DNA damage, epigenetic changes, aggregation of damaged proteins, and mitochondrial dysfunction [78]. These stresses lead to a reduction in differentiation potential and proliferation capacity [65, 79]. In order to adapt to cellular damage, cells are regulated through stress response pathways such as protein homeostasis, DNA damage repair, and mitochondrial respiratory metabolism, but these mechanisms become progressively dysregulated during senescence [80, 81]. It has been found that by regulating signaling pathways such as Notch, TGF‐β, JAK/STAT, p38 MAPK, sirtuins, oxytocin, and mTOR, it may be possible to alter the senescence phenotype of stem cells [77].

**FIGURE 2:**
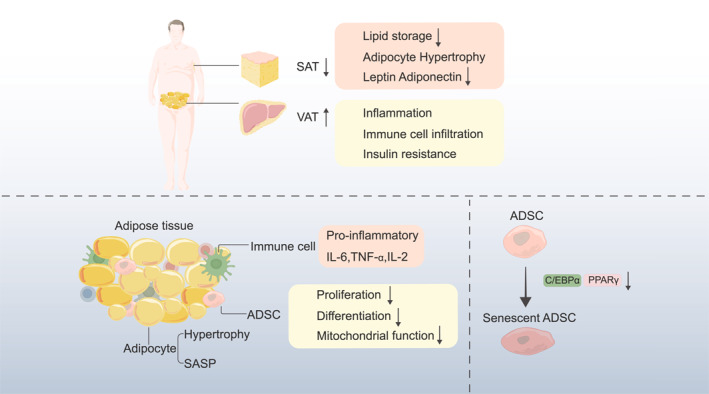
Age‐associated dysfunction of adipose depots and ADSCs. Aging leads to a progressive decline in both SAT and increase in VAT, accompanied by reduced lipid storage capacity, adipocyte hypertrophy, and decreased levels of key adipokines such as leptin and adiponectin. VAT aging is further characterized by chronic low‐grade inflammation, increased immune‐cell infiltration, and insulin resistance. Within adipose tissue, aged adipocytes exhibit hypertrophy and a SASP, producing elevated levels of pro‐inflammatory cytokines including IL‐6, TNF‐α, and IL‐2, which reshape the immune milieu and impair tissue homeostasis. Meanwhile, ADSCs progressively lose their regenerative competence, showing diminished proliferation, reduced differentiation potential, and compromised mitochondrial function. These alterations are accompanied by downregulation of adipogenic transcription factors such as C/EBPα and PPARγ, ultimately driving ADSC senescence and contributing to impaired adipose tissue remodeling during aging.

ADSCs derived from obese individuals showed reduced mitochondrial content compared with normal individuals, in addition to exhibiting general senescence phenotypes [82]. In another study in senescent ADSCs, there was a decrease in mitochondrial matrix density, impaired membrane potential, decreased fatty acid metabolites, and increased superoxide [83, 84]. Mitochondrial function is critical for maintaining the balance between stem cell self‐renewal and differentiation. Mitochondrial dysfunction disrupts the fine balance of metabolic pathways and signaling networks, leading to altered differentiation outcomes [85] (Figure 2).

## 3 Mitochondrial Factors in ADSC Senescence

### 3.1 Mitochondria Function

Mitochondrial dysfunction is widely recognized as a core driver of the aging process [86, 87]. Beyond serving as the primary cellular source of energy, mitochondria are essential regulators of cellular metabolism, apoptosis, calcium homeostasis, and reactive oxygen species (ROS) balance. According to the mitochondrial free radical theory of aging, ROS generated as byproducts of aerobic metabolism possess high chemical reactivity and can induce oxidative damage to lipids, proteins, and nucleic acids [88]. During aging, progressive mitochondrial impairment leads to excessive ROS production, accompanied by a gradual decline in the activity of antioxidant defense enzymes. In parallel, mutations in mitochondrial DNA (mtDNA) accumulate over time and are thought to actively contribute to aging‐associated functional deterioration [89]. Moreover, mitochondrial activity is tightly integrated with intracellular signaling pathways, and alterations in mitochondrial metabolism and biosynthesis are closely linked to SASP [90, 91]. In ADSCs, intact mitochondrial function is indispensable for maintaining cellular homeostasis. Mitochondrial dysfunction in ADSCs, manifested by reduced ATP generation, elevated ROS levels, disrupted mitochondrial dynamics, and compromised mtDNA integrity, results in impaired self‐renewal capacity, diminished differentiation potential, and attenuated regenerative function [92, 93].

### 3.2 Mitochondria, Inflammation and the SASP

mtDNA mutations also occur in senescent cells. mtDNA is susceptible to the effects of senescence due to its high replication rate, limited repair capacity, oxidative microenvironment, and lack of protective histones. Oxidative damage, replication errors, and insufficient repair mechanisms are major factors in the accumulation of mtDNA mutations [94, 95, 96]. mtDNA mutations can lead to electron transport chain (ETC) dysfunction, decrease ATP production and increase ROS production, which further exacerbates oxidative stress and mtDNA damage in a vicious cycle [97]. In ADSCs, mtDNA mutations impair regenerative potential by impairing their self‐renewal and differentiation capacity [85, 98].

In addition, intracellular mitochondria have a set of quality control mechanisms to ensure normal function and integrity, including mitochondrial generation, fusion and division dynamics, and removal of damaged or excess mitochondria through mitochondrial autophagy [99, 100, 101]. Studies have shown that cellular senescence is associated with altered mitochondrial dynamics, with elongated, enlarged, and hyperfused mitochondria (hyperfusion) common in senescent cells [102, 103]. Mitochondrial autophagy is a form of autophagy that specializes in the removal of damaged or excess mitochondria. However, mitochondrial autophagy is impaired in senescent cells. It has been shown that impaired mitochondrial autophagy promotes cellular senescence, whereas enhanced mitochondrial autophagy allows for functional restoration of mitochondria and maintenance of stem cell stemness, thereby supporting tissue regeneration [104].

Other studies have shown that mitochondrial function is strongly associated with SASP, and senescent cells with removed mitochondria exhibit a significantly reduced senescent secretory phenotype. Mitochondrial dysfunction can trigger inflammation through a variety of mechanisms, including mtDNA release, ATP release, N‐formylated peptide leakage, and ROS overproduction [105, 106].

### 3.3 Mitochondrial Dynamics and Quality Control

**FIGURE 3:**
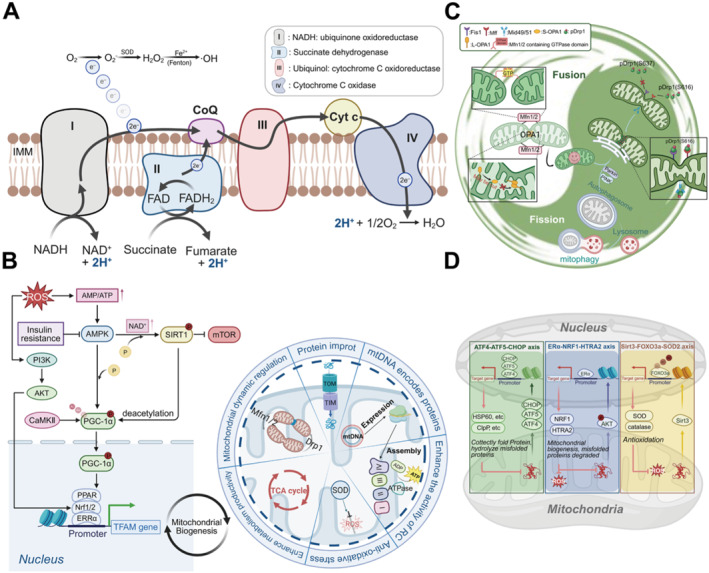
Mitochondrial electron transport, signaling networks, dynamics, and stress responses that regulate mitochondrial homeostasis. (A) ETC and oxidative phosphorylation. Schematic representation of complexes I–IV in the mitochondrial inner membrane, illustrating electron transfer from NADH and FADH_2_ to ubiquinone and cytochrome c, ultimately driving proton translocation and ATP production. Leakage of electrons, particularly from complexes I and III, generates superoxide (O_2_·^−^), which can be dismutated to H_2_O_2_ and further converted to hydroxyl radicals through Fenton chemistry, contributing to mitochondrial oxidative stress. (B) Mitochondrial biogenesis and metabolic signaling pathways. ROS accumulation and insulin resistance activate AMPK and SIRT1 in response to altered AMP/ATP and NAD^+^ levels, whereas mTOR and PI3K–AKT modulate downstream mitochondrial programs. Activated PGC‐1α interacts with nuclear transcription factors (PPAR, NRF1/2, ERRα) to promote transcription of mitochondrial genes such as TFAM, thereby enhancing mitochondrial DNA replication, protein import, assembly of respiratory complexes, and overall mitochondrial biogenesis. (C) Mitochondrial dynamics: fusion, fission, and mitophagy. Mitochondrial morphology is regulated by fusion proteins and fission machinery driven by Drp1 activation through phosphorylation and inhibition. Excessive fission promotes mitochondrial fragmentation and mitophagy, whereas coordinated fusion supports ATP production and mtDNA integrity. Dysregulated fusion–fission balance contributes to mitochondrial dysfunction under stress. (D) Mitochondrial stress response and mito–nuclear communication. Three major axes of the UPR^mt^ are shown: ATF4/ATF5–CHOP, ERα–NRF1–HTRA2, and Sirt3–FOXO3a–SOD2 pathways. These signaling networks enhance mitochondrial proteostasis, antioxidant defenses, and transcriptional reprogramming in the nuclear to maintain mitochondrial function. Modified and reprinted under terms of the CC‐BY license [114]. Copyright 2025, The Authors, published by John Wiley and Sons.

Mitochondria are highly dynamic organelles that are able to flexibly switch between oxidative phosphorylation (OXPHOS) and glycolysis based on nutrient availability and cellular demand [107, 108]. When stem cells enter the senescent state, their AMP/ATP and ADP/ATP ratios increase. This decrease in the efficiency of ATP production is associated with a decrease in mitochondrial membrane potential and a decrease in the degree of OXPHOS [109, 110]. Mitochondrial metabolic imbalance activates the AMPK pathway, which further induces p53 phosphorylation, up‐regulation of p16 expression, and reduced phosphorylation of Rb proteins, thereby promoting cell cycle arrest and senescence phenotypes [111, 112]. Senescent cells usually exhibit dysregulated OXPHOS producing excessive ROS that can induce cellular senescence via the DNA damage response (DDR) and p53/p21 pathway [113] (Figure 3).

## 4 Nuclear–Mitochondria Communication in ADSCs

### 4.1 Mechanisms of Nuclear–Mitochondria Communication in ADSCs Senescence

Multiple organelles in cells are connected. Mitochondria and nuclear communication is one of the key parts [115]. There is bidirectional communication between mitochondria and the nuclear, which will be discussed in detail in a later section. In summary, the nuclear, through anterograde signal, can regulate mitochondrial function, and the mitochondria, through a retrograde signal, can regulate the nuclear [116]. Anterograde signals can promote mitochondrial biosynthesis or enhance mitochondrial activity in response to cellular demands. This regulation can induce the expression of mitochondrial DNA‐encoded genes through mitochondrial transcription factor (TFAM), peroxisome proliferator‐activated receptor gamma coactivator 1α (PCG1α), and nuclear respiratory factor 1 (Nrf1) [117]. However, in senescent cells, due to cumulative damage to the nuclear genome, epigenetic modifications are altered and the accessibility of transcription factors and other regulatory molecules to DNA is abnormal, thus affecting positive communication [118]. Retrograde signal is often a process that transmits signals to the nuclear when mitochondria become dysfunctional. For example, a range of tricarboxylic acid cycle (TCA) metabolites can mediate retrograde signals. These metabolites are involved in epigenetic modifications and chromatin remodeling, regulating histone acetylation, methylation, and key enzymes of DNA methylation [119].

### 4.2 Nuclear‐to‐Mitochondria (Anterograde) Communication

Most of the mitochondrial proteome is encoded by cytosolic genes. Thus, expression of the mitochondrial proteome requires close coordination between the nuclear and the mitochondrial genomes [120]. In mitochondrial homeostasis and stress, a number of proteins and signaling pathways mediate nuclear‐mitochondria communication, where communication from the nuclear to the mitochondria is referred to as anterograde signal, and vice versa, communication from the mitochondria to the nuclear is referred to as retrograde signal. The nuclear system regulates mitochondria through anterograde signal in order to adjust mitochondrial activity and biogenesis to cellular needs. At the same time, reverse communication is sent when mitochondria are damaged to adapt to cellular functions and repair mitochondrial problems by altering nuclear gene expression, resulting in a bidirectional system of “mitochondrial‐nuclear communication.” This communication ensures homeostasis and adaptive capacity by integrating responses under normal and stressful conditions through mitochondrial‐nuclear feedback. When stress is more severe, the integration stress response (ISR) may be triggered, which reduces protein synthesis or triggers non‐cell‐autonomous responses, which modulate distant cellular functions to facilitate the organism's adaptation to stress [117].

The regulation of nuclear‐encoded mitochondrial proteins consists of two key transcription factors, nuclear respiratory factor (NRF1 and GABPα/NRF2α). These two transcription factors, especially NRF1, regulate the transcription of cytochrome c and most nuclear‐encoded OXPHOS‐related genes. NRF1 also regulates mtDNA replication, transcription and translation‐related protein expression. In addition, gene expression of mitochondrial ribosomal proteins, tRNA synthetases, and other genes is also regulated by NRF1 [121, 122]. Some cellular nuclear receptors can activate the expression of nuclear‐encoded mitochondrial proteins, such as peroxisome proliferator‐activated receptors (PPARs) that stimulate the expression of mitochondrial fatty acid oxidation‐associated enzymes in the heart and muscle [123] and estrogen‐related receptors (ERRs) [124]. These receptors correlate with the expression of nuclear‐encoded mitochondrial proteins associated with the TCA cycle, OXPHOS, and fatty acid oxidation.

The expression of mitochondrial‐encoded proteins is also regulated by the nuclear element, which encodes almost all factors that activate mitochondrial transcription and translation [125]. Activation of this anterograde signal is often dependent on upstream signaling. Changes in some metabolic conditions (e.g., reduced ATP synthesis) can activate PGC1α via AMPK and SIRT1, which in turn promotes mitochondrial energy metabolism and biogenesis. Increased calcium ions or cold stimulation, for example, also activate PGC1α through different pathways, thereby enhancing mitochondrial function [117]. In addition to anterograde protective regulation, some stress responses in the nuclear are also transmitted to mitochondria through positive communication; for example, nuclear DNA damage decreases mitochondrial metabolism and biogenesis by inhibiting PGC1α and PGC1β [126, 127].

**FIGURE 4:**
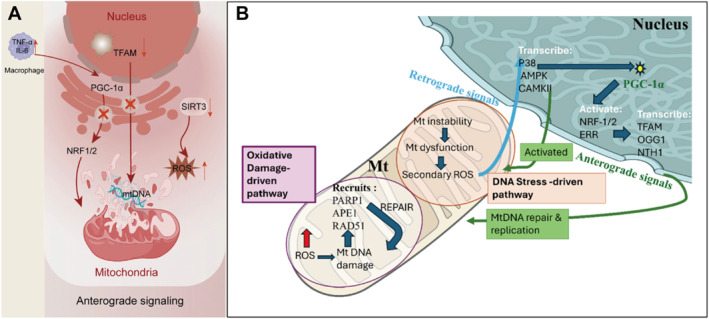
Bidirectional mito–nuclear communication during inflammation, oxidative stress, and mitochondrial DNA damage. (A) The defect in anterograde control diminishes mitochondrial gene expression and mtDNA maintenance, leading to heightened ROS production and disruption of mitochondrial homeostasis. SIRT3‐mediated deacetylation pathways are also suppressed, further exacerbating oxidative stress and metabolic decline. Modified and reprinted with permission [129]. Copyright 2025, The Authors, published by Springer Nature. (B) mtDNA damage‐induced retrograde signaling and coordinated repair responses. Oxidative stress and elevated ROS induce mtDNA lesions, mtDNA instability, and secondary mitochondrial dysfunction. Damaged mitochondria activate an oxidative‐damage pathway that recruits DNA repair factors including PARP1, APE1, and RAD51 to the organelle. Concurrently, mtDNA stress triggers retrograde signals that activate nuclear stress‐response pathways such as p38 MAPK, AMPK, and CaMKII, thereby stimulating PGC‐1α. Modified and reprinted under terms of the CC‐BY license [130]. Copyright 2025, The Authors, published by John Wiley and Sons.

In addition, some proteins can be translocated from the nuclear to mitochondria under oxidative stress. This is also a form of positive nuclear‐mitochondrial communication. When a cell is threatened by oxidative stress due to mitochondrial dysfunction, some proteins can be transferred from the nuclear to the cytoplasm under the action of nuclear exiting sequences (NES), and then from the cytoplasm to the mitochondria under the action of mitochondria targeting sequences (MTS) to play a role in cellular repair. Among them, Telomerase Reverse Transcriptase (TERT) is the protein with this function. When cells are subjected to oxidative stress, TERT can be transferred from the nuclear to the mitochondria to repair mitochondrial function, thus restoring the physiological function of cells [128] (Figure 4).

### 4.3 Mitochondria‐to‐Nuclear (Retrograde) Communication

In contrast to anterograde signals, mitochondria can also regulate cellular and organismal activities through a variety of retrograde signals and prevent mitochondrial dysfunction by activating metabolic reprogramming or the expression of stress‐defense‐related nuclear genes. The major ones include energy stress response, calcium‐dependent response, reactive oxygen stress response, and mitochondrial proteostasis response [131].

**FIGURE 5:**
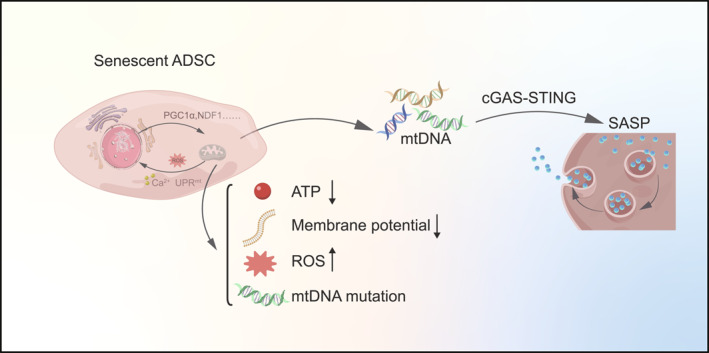
Mitochondrial dysfunction–induced mtDNA release activates cGAS–STING signaling and SASP production in senescent ADSCs. Senescent ADSCs exhibit impaired mitochondrial homeostasis characterized by reduced expression of mitochondrial biogenesis regulators, altered Ca^2+^ handling, decreased membrane potential, and diminished ATP production. These defects increase mitochondrial ROS levels and promote the accumulation of mtDNA mutations. Damaged or destabilized mitochondria release mtDNA into the cytosol, where it is sensed by the cGAS–STING pathway. Activation of cGAS–STING drives transcription of pro‐inflammatory genes and secretion of SASP factors, thereby amplifying inflammatory signaling and reinforcing the senescent cell state.

Studies of the energetic stress response have been conducted mainly in yeast and nematodes [132, 133]. In mammals, this response is mainly mediated through the AMPK and mTOR pathways. AMPK was mentioned in the previous section as an upstream signaling pathway for positive communication. When mitochondrial ATP synthesis is reduced, it activates AMPK, which promotes PGC1α activation and enhances mitochondrial energy metabolism and biogenesis [134]. AMPK also initiates the mitochondrial quality control system, regulates mitochondrial dynamics and induces mitochondrial autophagy [135]. The mTOR pathway is also involved in retrograde signals. mTOR activity inhibits retrograde signaling when elevated, whereas under energetic stress (e.g., exercise or nutritional restriction), mTOR activity is reduced, which contributes to retrograde signaling [136]. In addition, when mitochondria are stressed, the mitochondrial membrane potential is lost, resulting in the release of Ca^2+^ into the cytoplasm [137]. Ca^2+^ activates transcription factors in two ways: one is that calcineurin is activated and activates transcription factors such as the NF‐κB p105 subunit, which are translocated to the nuclear to promote the synthesis of proteins related to Ca^2+^ transport and storage as well as glycolytic and glycolytic enzymes [117]. The other is the direct activation of multiple Ca^2+^‐dependent kinases, including CAMKIV, Ca^2+^‐dependent protein kinase C, JNK, and p38 MAPK, when free Ca^2+^ levels are elevated, which ultimately stimulates the activation of multiple transcription factors (e.g., CREB, EGR1, ATF2,CEBPδ, and CHOP) activation [117]. ROS is also an important retrograde signal. When mitochondria are dysfunctional, large amounts of ROS are produced due to ETC defects. ROS can induce the expression of detoxifying enzymes and antioxidant proteins via transcription factors (e.g., NFE2L2/NRF2) in combination with antioxidant response elements (ARE) [119]. ROS can also activate the NF‐κB pathway. ROS can also regulate metabolic reprogramming and AMPK activation through the JNK‐PGC1α pathway by inducing mitochondrial biogenesis and OXPHOS‐related gene expression [138, 139, 140]. The most important aspect of nuclear‐mitochondrial retrograde signal is the maintenance of mitochondrial protein homeostasis. Mitochondria possess a conserved protein quality control system consisting mainly of nuclear‐encoded molecular chaperones and proteases. These proteins are responsible for the folding, assembly and turnover of mitochondrial proteins and maintain mitochondrial protein homeostasis under normal and stress conditions. When stress is dysregulated, mitochondria communicate with the nuclear through signaling to promote the expression of quality control components and other compensatory genes to restore mitochondrial homeostasis. Among these, the mitochondrial unfolded protein response, UPR^mt^, can activate the expression of proteases, molecular chaperones, and other stress‐responsive genes [117, 119] (Figure 5).

### 4.4 Nuclear–Mitochondria Communication Regulates ADSCs Senescence

Mitochondrial stress can initiate ADSC senescence through multiple, partial convergent routes. First, elevated mtROS promotes persistent DDR signaling, which cooperates with NF‐κB and C/EBPβ to drive canonical SASP transcriptional programs [141]. Second, mitochondrial membrane perturbation can lead to cytosolic leakage of mtDNA and other mitochondrial nucleic acids, thereby reinforcing inflammatory SASP outputs [90]. Third, mitochondrial dysfunction often causes NAD^+^ depletion and altered NAD^+^/NADH redox balance, suppressing sirtuin activities and facilitating hyper‐acetylation of transcriptional regulators that favor senescence and SASP [142]. Fourth, mitochondrial Ca^2+^ handling defects can activate Ca^2+^‐dependent phosphatases, which intersects with pro‐inflammatory transcriptional circuits [143]. In parallel, mitochondrial proteostasis stress activates the mitochondrial unfolded protein response via ATF4/ATF5/CHOP, reshaping nuclear transcription and metabolic remodeling that can synergize with SASP programs under chronic stress [144]. Beyond intracellular retrograde signals, mitochondrial stress also elicits secreted “mitokines” that can act in an autocrine/paracrine manner to propagate a senescence‐permissive microenvironment [145]. Among reported mitokines, GDF15 and FGF21 are induced by mitochondrial stress‐responsive transcription factors and have been implicated in systemic inflammatory adaptation [146].

## 5 Latest Nuclear‐Mitochondria Crosstalk Based Adipose Senescent Therapeutic Strategies

### 5.1 Strategies for Targeting Adipose Tissue

In order to precisely regulate adipose tissue function, we need a series of methods to precisely target adipose tissue for gene delivery or other interventions. The targeting of adipose tissue is mainly divided into active targeting and passive targeting. Passive targeting mainly refers to the obese adipose tissue, due to the formation of new blood vessels and newborn adipocytes, coupled with the chronic inflammatory environment, macrophages, leukocytes infiltration increases vascular permeability, which promotes the nanoparticles passive targeting to adipose tissue [147, 148]. However, passive targeting lacks specificity, so a large number of studies have begun to focus on finding ways to actively target adipose tissue. It has been reported that some short peptides can be targeted to adipocytes, such as CKGGRAKDC, which can specifically bind to inhibition (PHB) on vascular endothelial cells, white adipocytes [149, 150]. In addition, aptamers (adipo) act as antibody‐like DNA or RNA molecules that bind with high specificity and affinity to differentiate mature adipocytes [151]. However, these short peptides or aptamers often need to be coupled with liposomes to accomplish gene delivery, posing difficulties for simple and convenient adipose tissue gene delivery.

In 2022, it was reported that the third‐generation dendrimer PAMAM (PAMAM generation 3, PG3) has good adipose targeting properties, and can be widely distributed to adipose tissues carrying glycosaminoglycosides due to its positive charge. In particular, it can be distributed to adipose stromal cells [152, 153]. PG3 is also a good gene delivery vector. Meanwhile PG3 is also a good gene delivery vector [154]. Therefore, using PG3 as a gene delivery vehicle, it may be possible to achieve gene delivery to adipose tissue mechanism cells.

In addition to targeting adipose tissue. Specific targeting of adipose stromal cells such as ADSCs can more precisely regulate their related functions. Currently, there are relatively few studies on targeting ADSCs. It is known that glycosylation site‐deficient decorin (ΔDCN) is distributed in both mouse and human ADSCs, and the peptide CSWKYWFGEC, a cysteine‐rich cyclic peptide library, binds specifically to ΔDCN [155, 156]. Thus, modification of this peptide on liposomes may enable precise regulation of ADSCs.

### 5.2 Regulation of Metabolic Homeostasis in Adipose Tissue

Our group recently focused on the nuclear‐mitochondria anterograde signal mediated by TERT. In our research, we explored the potential of TERT as a crucial mediator in the repair of mitochondrial function, particularly in the context of ADSC senescence. We developed a novel mito‐TERT activation strategy, leveraging a gene/fiber‐plex system designed for precise targeting of senescent ADSCs. Our findings demonstrated that the mito‐TERT significantly improved mitochondrial function in senescent ADSCs, thus reversing the detrimental effects of senescence in adipose tissues. We used PG3‐TERT@CoQ10, a composite formulation that combines TERT plasmids with Coenzyme Q10 to transiently modulate oxidative stress, facilitating the activation of mito‐TERT. This activation process involved reducing intracellular ROS levels, promoting TERT translocation from the nuclear to the mitochondria, and enhancing mitochondrial DNA repair and function. In vitro and in vivo experiments revealed that the PG3‐TERT@CoQ10 gene/fiber‐plexes were highly effective in reversing adipose senescence, leading to improved metabolic stability and adipose tissue homeostasis in obese mice. Furthermore, our research indicates that targeting the regulation of mitochondrial‐nuclear retrograde communication (UPR^mt^) in senescent ADSCs shows positive effects. Therefore, the bidirectional communication between the nuclear and mitochondria plays a crucial and meaningful role in restoring the function of senescent ADSCs.

### 5.3 TERT‐Mediated Positive Nuclear‐Mitochondria Communication

**FIGURE 6:**
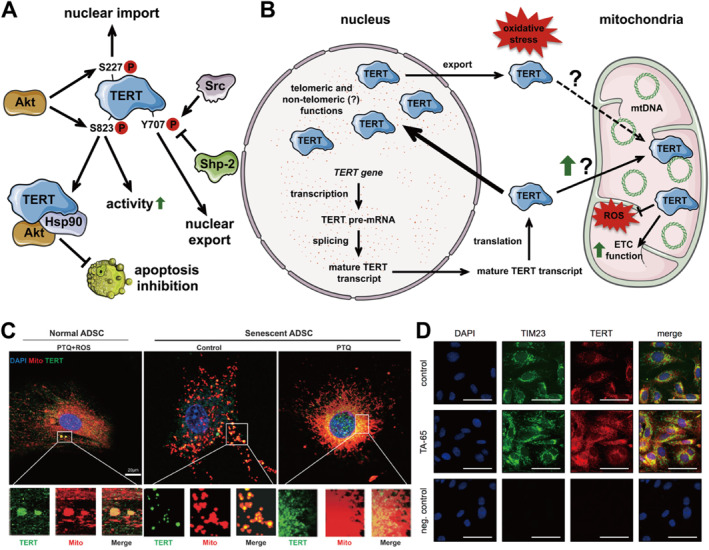
Regulation of TERT localization and mitochondrial functions in normal and senescent ADSCs. (A) TERT trafficking is controlled by phosphorylation at key residues, Akt‐mediated phosphorylation at S227 promotes nuclear import, while S823 phosphorylation enhances enzymatic activity. Conversely, Src‐mediated phosphorylation at Y707 facilitates TERT nuclear export. The TERT–Hsp90–Akt complex stabilizes TERT and inhibits apoptosis. These combinatorial post‐translational modifications orchestrate the dynamic shuttling of TERT between cellular compartments. (B) Under homeostatic conditions, the TERT gene undergoes transcription, pre‐mRNA splicing, and translation to generate mature TERT protein, primarily functioning within the nuclear. Oxidative stress triggers TERT export and redistribution toward mitochondria, where TERT may mitigate mtDNA damage, support ETC activity, and reduce mitochondrial ROS generation. Although incompletely understood, mitochondrial TERT is proposed to participate in maintaining mtDNA integrity and enhancing cellular stress resistance. Modified and reprinted with permission [128]. Copyright 2020, The Authors, published by Elsevier. (C) Representative images show TERT (green), mitochondria (red), and nuclei (DAPI) in normal ADSCs and senescent ADSCs with or without PTQ or PTQ + ROS treatment. In normal ADSCs, TERT partially colocalizes with mitochondria, whereas senescent ADSC exhibit diminished mitochondrial TERT signals and increased mitochondrial abnormalities. Magnified insets highlight the degree of TERT–mitochondria overlap under each condition. Modified and reprinted with permission [170]. Copyright 2025, John Wiley and Sons. (D) Immunostaining for TERT (red) and the mitochondrial inner membrane marker TIM23 (green) in control, TA‐65–treated, and negative control ADSCs. TA‐65 markedly increases mitochondrial TERT localization, while the negative control shows minimal signal. Modified and reprinted with permission [168]. Copyright 2021, Wolters Kluwer Health, Inc.

Telomeres are located at the ends of chromosomes and consist of repetitive hexanucleotide sequences (5′‐TTAGGG‐3′) and single‐stranded protruding sequences that bind specific proteins to form unique structures [157]. The single‐stranded portion of telomeres is inserted into adjacent DNA double‐stranded strands to prevent chromosome ends from being mistaken for DNA damage, thus protecting the integrity and stability of chromosomes. The length of telomeres shortens as the organism ages, with human telomeres being about 11 kb in length at birth, and due to the end‐replication problem of DNA replication, telomeres shorten at each cell division, reaching a critical length that triggers cellular senescence [158]. The shortening of telomeres can be reversed by telomerase, a ribonucleoprotein consisting of a catalytic subunit (TERT) and an RNA component (Telomerase RNA Component, TERC). The TERC binds to the telomere single strand as a template, and the TERT carries out the reverse transcription by adding hexanucleotide repeat sequences. Telomerase activity requires the involvement of other accessory proteins [159]. TERT and TERC are required for the maintenance of telomere function, with TERC being recognized as a major limiting factor for telomerase activity [160]. TERT is normally expressed in cells with high replication capacity, thus preventing telomere shortening. However, recent studies have shown that TERT is also found in tissues with low replication capacity, such as blood vessels, heart and brain [160]. Post‐translational regulation of TERT is important for its localization and function, and phosphorylation at serine 823 activates TERT and promotes its intranuclear localization by forming a stable complex with Akt and Hsp90 [161]. Meanwhile, phosphorylation of the serine 227 site is essential for TERT nuclear import [162]. Phosphorylation at tyrosine 707 leads to extra‐nuclear transport of TERT; this process can be inhibited by Shp‐2 phosphatase. Phosphorylation at the tyrosine 707 site leads to extra‐nuclear translocation of TERT; this process can be inhibited by Shp‐2 phosphatase [163]. Oxidative stress is induced when there is an intracellular redox imbalance. It has been found that oxidative stress promotes the transport of TERT tyrosine 707 from the nuclear to the mitochondria by phosphorylating the TERT tyrosine 707 site via the Src kinase family [164, 165]. TERT that enters the mitochondria binds to mitochondrial DNA (mtDNA) and tRNA and accomplishes positive communication from the nuclear to the mitochondria. TERT protects mitochondrial DNA (mtDNA), reduces ROS production, improves the mitochondrial membrane potential and increases the level of manganese superoxide dismutase (MnSOD) [166, 167, 168, 169, 170]. Thus, the translocation of TERT from the nuclear to the mitochondria in response to cellular oxidative stress is a mode of regulation of positive nuclear‐mitochondria communication, but this set of repair mechanisms is impaired in senescent ADSCs and will be discussed in detail in subsequent sections (Figure 6).

### 5.4 UPR^mt^‐Mediated Nuclear‐Mitochondria Retrograde Signal

The UPR^mt^ response, as a retrograde signal to the nuclear under mitochondrial stress, is a protective transcriptional response that maintains mitochondrial function and adapts to the stressful environment by enhancing the expression of mitochondrial proteostatic and metabolic genes. Predisposing factors include proteotoxic stress (accumulation of unfolded proteins, impaired quality control systems), mitochondrial‐nuclear imbalance and inhibition of ETC function [171]. The UPR^mt^ reaction is well‐studied in the cryptic nematode *Caenorhabditis elegans* and is mediated by the key transcription factor ATFS‐1, which has mitochondrial targeting sequences and nuclear localization signals. Under normal conditions, it continuously enters mitochondria and is degraded by LON protease (LONP).In contrast, under stress conditions ATFS‐1 is translocated from mitochondria to the nuclear, where it synergizes with DVE‐1 and UBL‐5 to induce the expression of several mitochondrial quality control genes (e.g., HSP60, DNJ‐10, DRP‐1, and components of the TIM23 complex), as well as repressing genes encoding the TCA cycle and ETC subunits [119]. The UPR^mt^ response has also been partially studied in Drosophila (*Drosophila melanogaster*), where dysregulation of intracellular mitochondrial protein homeostasis in Drosophila cells in the presence of overexpression of the mutant mitochondrial protein mitochondrial ornithine carbamoyltransferase (ΔOTC) further induces HSP60 and mtHSP70 expression [172]. Furthermore, mild mitochondrial damage caused by specific knockdown of the mitochondrial complex I component Nd75 in Drosophila muscle cells not only triggers UPR^mt^ but also activates the mitochondrial adaptive response (mitohormesis) [173]. Several studies have shown that the UPR^mt^ response is conserved in mammals, and in monkey COS7 cells, ΔOTC‐induced dysregulation of proteostasis can lead to the activation of CHOP and CEBPβ, inducing HSP60, HSP10 and other UPR^mt^ Gene expression [117]. In mouse skeletal muscle and cardiac myocytes, aspartyl tRNA synthetase deficiency leads to loss of mitochondrial proteostasis and activation of the UPR^mt^ response in the heart and promotes mitochondrial biosynthesis [174]. Specific knockdown of the mitochondrial fusion protein mitofusins in mouse cardiomyocytes hinders mitochondrial fusion, leads to accumulation of damaged mitochondria, and induces the UPR^mt^ response [175]. Twinkle mitochondrial deconjugase mutations lead to multiple mtDNA deletions and late‐onset mitochondrial disease in mice, accompanied by defects in OXPHOS function and up‐regulation of HSP60, mtHSP70, and CLPP protein levels [176]. UPR^mt^ not only regulates mitochondrial proteostasis, but also promotes the regenerative capacity of hematopoietic stem cells through SIRT7 and NRF1, and interacts with other quality control systems such as mitochondrial autophagy and antioxidant response [177].

**FIGURE 7:**
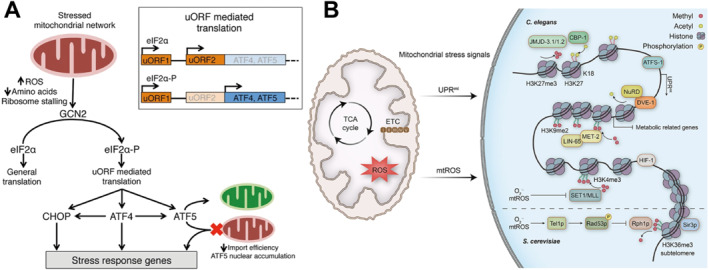
Mitochondrial stress–induced translational rewiring and epigenetic regulation of the UPR^mt^. (A) Mitochondrial dysfunction characterized by elevated ROS levels, amino acid imbalance, and ribosome stalling activates GCN2, leading to phosphorylation of eIF2α. eIF2α phosphorylation suppresses global protein synthesis while selectively enhancing translation of ATF4 and ATF5 through uORF‐dependent mechanisms. ATF4 and ATF5 cooperate to induce stress‐response transcriptional programs, including CHOP activation. Under mitochondrial stress, impaired mitochondrial import promotes nuclear accumulation of ATF5, amplifying transcriptional induction of mitochondrial stress response genes and contributing to the ISR. (B) Mitochondrial ROS triggers organism‐specific chromatin remodeling to activate UPR^mt^. Mitochondrial ROS and ETC‐derived stress signals activate the UPR^mt^ through conserved epigenetic pathways. In *Caenorhabditis elegans*, UPR^mt^ activation involves histone demethylases, CBP‐1–mediated acetylation, and NuRD‐driven chromatin remodeling to induce metabolic and proteostasis‐related genes via ATFS‐1. In contrast, in *Saccharomyces cerevisiae*, mtROS promotes histone modifications including SET1/MLL‐mediated H3K4 methylation, Sir3p‐associated chromatin repression, and subtelomeric remodeling to regulate stress adaptation. Modified and reprinted under terms of the CC‐BY license [171]. Copyright 2018, The Authors, published by Springer Nature.

In addition, the UPR^mt^ response that can be generated by the cell itself, it can also be transmitted to other cells through non‐autonomous responses. Mitochondrial stress triggers extracellular signals characterized by the secretion of mitochondrial cytokines (mitokines) [116]. In the hidradenitis elegans nematode, mitochondrial stress signaling triggered by selective knockdown of ETC subunits (e.g., cco‐1) in neurons not only activates the UPR^mt^ within the nervous system, but also transmits to distal tissues, such as the intestinal tract, and it beneficially affects the whole organism and significantly prolongs the lifespan of the organism [178]. In addition, minor ETC defects in *Drosophila* muscle can be transmitted to other tissues via insulin signaling, and these sources of cross‐tissue signaling are critical for longevity [179]. In mammals, mitochondria‐mediated non‐autocytotic responses may be regulated by mitochondria‐derived peptides or cytokines. Some mitochondria‐derived peptides are produced by open reading frames (ORFs) encoded by mitochondrial DNA [180]. For example, humanin, encoded by the 16S rRNA gene, is capable of being secreted from mitochondria and exhibits cytoprotective effects in Alzheimer's disease models [181]. FGF21 is a fasting‐regulated hormone with pleiotropic effects in different tissues. FGF21 is secreted in OXPHOS‐deficient skeletal muscle and is a potent mitokine [182]. In conclusion, UPR^mt^ exerts a beneficial protective effect on cells through nuclear‐mitochondrial reverse communication; however, this response is downregulated in senescent cells [183, 184]. Therefore, how to modulate nuclear‐mitochondria reverse communication in senescent ADSCs and reactivate this response may be able to restore their mitochondrial function (Figure 7).

## 6 Emerging Organelle‐ and Genome‐Engineering Strategies for ADSC Rejuvenation

### 6.1 Mitochondrial Transfer

Mitochondrial transfer has become a research hotspot in recent years and may achieve rejuvenation of senescent adipose‐derived stem cells (ADSCs) by directly supplementing functional mitochondria [185]. Intercellular mitochondrial transfer can occur through tunneling nanotubes, microvesicles, or cell fusion, and engineered approaches have now enabled the controlled delivery of isolated mitochondria into target cells [186]. In senescent ADSCs, supplementation with exogenous mitochondria can improve cellular mitochondrial function [187].

### 6.2 Mitochondrial Gene Editing

Although mitochondrial transplantation has certain therapeutic potential, directly editing the mitochondrial genome is a more precise and effective approach. It can be used to edit mtDNA mutations and transcriptional defects that accumulate during ADSC senescence [188]. Unlike nuclear genome editing, mtDNA lacks the canonical double‐strand break repair pathway, which renders traditional CRISPR/Cas9 approaches ineffective in mitochondria [189]. However, recent advances have made it possible to achieve targeted modification of mtDNA through novel editing systems, such as DdCBE (DddA‐derived cytosine base editors), TALE‐linked deaminases, and zinc‐finger nucleases, which are capable of selectively eliminating mutant mitochondrial genomes through heteroplasmy shifting [190]. These tools enable precise C•G → T•A base conversion or targeted degradation of dysfunctional mtDNA species, thereby restoring respiratory chain function and reducing ROS‐driven retrograde stress signaling [190].

### 6.3 Mitochondria‐Targeted Gene/Protein Delivery

In addition, the direct delivery of exogenous genes or proteins into mitochondria has also become a major research focus [191]. Unlike whole‐organelle transplantation or genome editing, this strategy delivers functional nucleic acids or proteins directly into mitochondria to compensate for impaired endogenous expression [192]. Mitochondrial targeting is usually achieved by conjugating therapeutic cargos with mitochondrial targeting sequences (MTS), lipophilic cations, or mitochondria‐penetrating peptides (MPPs), thereby promoting efficient translocation across mitochondrial membranes [193]. In recent years, nanoparticle platforms, including MTS‐liposomes, mitochondria‐tropic polymers, and dendrimer‐based delivery systems, have further expanded the ability to deliver plasmids, mRNA, or recombinant proteins with high specificity and low cytotoxicity [194]. In senescent ADSCs, mitochondria‐targeted delivery of regulatory proteins such as TFAM or SOD2 can enhance mtDNA stability, refold misfolded OXPHOS proteins, and reduce ROS‐mediated retrograde inflammation. Similarly, delivery of PGC‐1α or mito‐TERT expression constructs can enhance mitochondrial biogenesis, repair oxidative mtDNA damage, and restore membrane potential [195].

### 6.4 Mitochondrial Extracellular Vesicle Delivery

An emerging direction in mitochondrial engineering is the application of Mito‐EVs. Unlike synthetic nanoparticles or whole‐organelle transplantation, MitoEVs encapsulate intact mitochondria, mtDNA, and respiratory chain components within a vesicular membrane derived from donor cells, thereby providing a physiologically stable and immunologically tolerated delivery vehicle [196]. These vesicles can fuse with ADSCs through endocytosis or direct membrane fusion, thereby transferring functional mitochondria into cells with impaired mitochondrial function [197]. Engineered MitoEVs can further enhance targeting by incorporating targeting ligands, thus providing a customizable platform for precision mitochondrial augmentation [198] (Table 1).

**TABLE 1:** Comparison of emerging organelle‐ and genome‐engineering strategies for ADSC rejuvenation.

Strategy	What is delivered	Key benefits	Main limitations
Mitochondrial transfer	Isolated mitochondria	Fast organelle‐level rescue; broad applicability	Source/quality control, uptake efficiency, standardization; compatibility and immunologic issues [185, 186, 187]
Mitochondrial gene editing	mtDNA editors	High precision at genome level	Delivery to mitochondria, off‐target assessment, heteroplasmy control, long‐term safety [188, 189, 190]
Mito‐targeted gene/protein delivery	Mito‐targeted plasmid/mRNA/proteins	Modular cargos; rapid functional rescue; can bypass defective nuclear programs	Import efficiency and durability, dosing and carrier toxicity, manufacturing standardization [191, 192, 193, 194, 195]
Mito‐EV delivery (MitoEVs)	EVs carrying intact mitochondria, mtDNA, and respiratory components	Biocompatible, membrane‐protected cargo; potentially scalable; lower immunogenicity	Manufacturing; targeting specificity; batch heterogeneity and potency assays [196, 197, 198]

## 7 Conclusion and Outlook

In summary, mitochondrial dysfunction plays a critical role in the senescence of ADSCs and their subsequent impact on adipose tissue homeostasis. As we have discussed, the intricate communication between the nuclear and mitochondria, particularly the bidirectional signaling pathways, is essential for maintaining stem cell function and tissue regeneration. Disruptions to mitochondrial function, characterized by increased oxidative stress, mitochondrial DNA mutations, and impaired dynamics, exacerbate the senescent phenotype of ADSCs, diminishing their regenerative potential. Conventional strategies targeting mito‐nuclear crosstalk, such as enhancing TERT‐mediated mitochondrial repair or reactivating the UPR^mt^, provide meaningful opportunities to restore mitochondrial stability in aging ADSCs. However, the rapid emergence of organelle‐ and genome‐engineering technologies has expanded the therapeutic landscape far beyond traditional molecular interventions. Mitochondrial transplantation, synthetic mitochondria, mtDNA editing platforms such as DdCBE, mitochondria‐targeted gene/protein delivery systems, and MitoEV‐based mitochondrial augmentation represent transformative approaches capable of rebuilding mitochondrial function at the organelle, genome, or vesicular level. Together, these strategies offer unprecedented precision in repairing or replacing dysfunctional mitochondria and hold particular promise for re‐establishing healthy mito‐nuclear communication in senescent ADSCs. However, it is still not completely clear whether mito‐nuclear crosstalk functions as a primary driver of ADSC senescence or arises predominantly as a secondary consequence of upstream damage, and whether modulating these pathways can achieve functional rejuvenation, durable restoration of proliferation, differentiation capacity, and regenerative performance. Future research should focus on refining the mechanisms by which mitochondrial‐nuclear crosstalk can be modulated to specifically target senescent ADSCs. Given the heterogeneity of ADSCs, it will be important to determine whether specific ADSC subpopulations display distinct mitochondrial states and differential vulnerability to senescence. Key conclusions should be validated in primary human ADSCs from age‐stratified donors. Developing innovative gene delivery systems, such as those using PG3 dendrimer vectors, can improve the precision and efficiency of therapeutic interventions. Additionally, exploring the potential of combinatory approaches, such as targeting both mitochondrial function and cellular signaling pathways, could enhance therapeutic outcomes. Finally, understanding the role of mitokines in intercellular communication offers an exciting avenue for the development of novel therapies aimed at rejuvenating adipose tissue and combating age‐associated metabolic disorders.

## Author Contributions

**Yixiang Zhang:** investigation, writing – original draft. **Yawei Du:** investigation, writing – original draft. **Shifeng Ling:** investigation, writing – original draft. **Hanqi Wang:** investigation, writing – original draft. **Jiahao He:** visualization. **Jiahong Li:** visualization. **Kaifeng Huo:** visualization. **Baikun Liu:** data curation. **Qingfeng Li:** conceptualization, writing – review and editing. **Jiao Wei:** conceptualization. **Wenguo Cui:** conceptualization, writing – review and editing.

## Ethics Statement

The authors have nothing to report.

## Conflicts of Interest

The authors declare no conflicts of interest.

## Data Availability

The data that support the findings of this study are available from the corresponding author upon reasonable request.

## References

[1] Y. Qin , G. Ge , P. Yang , et al., “An Update on Adipose‐Derived Stem Cells for Regenerative Medicine: Where Challenge Meets Opportunity,” Advanced Science 10 (2023): 2207334. 37162248 10.1002/advs.202207334 PMC10369252

[2] L. K. Grun , R. M. Maurmann , J. N. Scholl , et al., “Obesity Drives Adipose‐Derived Stem Cells Into a Senescent and Dysfunctional Phenotype Associated With P38MAPK/NF‐KB Axis,” Immunity & Ageing 20 (2023): 51. 37821967 10.1186/s12979-023-00378-0 PMC10566105

[3] L. Scheja and J. Heeren , “The Endocrine Function of Adipose Tissues in Health and Cardiometabolic Disease,” Nature Reviews Endocrinology 15 (2019): 507–524. 10.1038/s41574-019-0230-6 31296970

[4] M. Y. Ou , H. Zhang , P. C. Tan , S. B. Zhou , and Q. F. Li , “Adipose Tissue Aging: Mechanisms and Therapeutic Implications,” Cell Death & Disease 13 (2022): 300. 35379822 10.1038/s41419-022-04752-6 PMC8980023

[5] Z. H. Yang , F. Chen , Y. Zhang , et al., “Therapeutic Targeting of White Adipose Tissue Metabolic Dysfunction in Obesity: Mechanisms and Opportunities,” MedComm 5 (2024): e560. 38812572 10.1002/mco2.560 PMC11134193

[6] M. Li , J. Li , X. Xiong , et al., “Heparinized PGA Host‐Guest Hydrogel Loaded With Paracrine Products From Electrically Stimulated Adipose‐Derived Mesenchymal Stem Cells for Enhanced Wound Repair,” Engineered Regeneration 4 (2023): 225–237.

[7] T. Lima , T. Y. Li , A. Mottis , and J. Auwerx , “Pleiotropic Effects of Mitochondria in Aging,” Nature Aging 2 (2022): 199–213. 37118378 10.1038/s43587-022-00191-2

[8] I. Bratic and A. Trifunovic , “Mitochondrial Energy Metabolism and Ageing,” Biochimica et Biophysica Acta (BBA) – Bioenergetics 1797 (2010): 961–967. 20064485 10.1016/j.bbabio.2010.01.004

[9] D. I. Savu and N. Moisoi , “Mitochondria – Nucleus Communication in Neurodegenerative Disease. Who Talks First, Who Talks Louder?,” Biochimica et Biophysica Acta (BBA) – Bioenergetics 1863 (2022): 148588. 35780856 10.1016/j.bbabio.2022.148588

[10] Y. X. Zhang , M. Y. Ou , Z. H. Yang , Y. Sun , Q. F. Li , and S. B. Zhou , “Adipose Tissue Aging Is Regulated by an Altered Immune System,” Frontiers in Immunology 14 (2023): 1125395. 36875140 10.3389/fimmu.2023.1125395 PMC9981968

[11] C. López‐Otín , M. A. Blasco , L. Partridge , M. Serrano , and G. Kroemer , “The Hallmarks of Aging,” Cell 153 (2013): 1194–1217. 23746838 10.1016/j.cell.2013.05.039 PMC3836174

[12] C. López‐Otín , M. A. Blasco , L. Partridge , M. Serrano , and G. Kroemer , “Hallmarks of Aging: An Expanding Universe,” Cell 186 (2023): 243–278. 36599349 10.1016/j.cell.2022.11.001

[13] X. Wu , H. Zhu , Y. Xu , B. Kong , and Q. Tan , “Chronic Wounds: Pathological Characteristics and Their Stem Cell‐Based Therapies,” Engineered Regeneration 4 (2023): 81–94.

[14] Z. Wang , W. Liang , R. Ao , and Y. An , “Adipose Decellularized Matrix: A Promising Skeletal Muscle Tissue Engineering Material for Volume Muscle Loss,” Biomaterials Research 29 (2025): 0174. 40248249 10.34133/bmr.0174 PMC12003953

[15] T. Cao , S. Liu , F. Wang , et al., “Insufficient Telomeric DNA Damage Response Promotes Chromosomal Instability in Aged Oocytes,” Science Bulletin 70 (2025): 3382–3396. 10.1016/j.scib.2025.08.034 40930920

[16] N. Schaum , B. Lehallier , O. Hahn , et al., “Ageing Hallmarks Exhibit Organ‐Specific Temporal Signatures,” Nature 583 (2020): 596–602. 32669715 10.1038/s41586-020-2499-y PMC7757734

[17] R. Pálovics , A. Keller , N. Schaum , et al., “Molecular Hallmarks of Heterochronic Parabiosis at Single‐Cell Resolution,” Nature 603 (2022): 309–314. 35236985 10.1038/s41586-022-04461-2 PMC9387403

[18] N. Almanzar , J. Antony , A. S. Baghel , et al., “A Single‐Cell Transcriptomic Atlas Characterizes Ageing Tissues in the Mouse,” Nature 583 (2020): 590–595. 32669714 10.1038/s41586-020-2496-1 PMC8240505

[19] D. Zhang , W. Liu , L. Feng , et al., “Innovative Advances in Droplet Microfluidics,” Research 8 (2025): 0856. 40881278 10.34133/research.0856 PMC12384975

[20] Q. Q. Tang , “Lipid Metabolism and Diseases,” Science Bulletin 61 (2016): 1471–1472.

[21] M. Blüher , “Fat Tissue and Long Life,” Obesity Facts 1 (2008): 176–182. 20054178 10.1159/000145930 PMC6452107

[22] S. Cinti , “The Endocrine Adipose Organ,” Reviews in Endocrine & Metabolic Disorders 23 (2022): 1–4. 35048260 10.1007/s11154-022-09709-w PMC8769372

[23] A. M. Cypess , “Reassessing Human Adipose Tissue,” New England Journal of Medicine 386 (2022): 768–779. 35196429 10.1056/NEJMra2032804

[24] M. Slawik and A. J. Vidal‐Puig , “Lipotoxicity, Overnutrition and Energy Metabolism in Aging,” Ageing Research Reviews 5 (2006): 144–164. 16630750 10.1016/j.arr.2006.03.004

[25] X. Fu , J. Wang , D. Qian , et al., “Living Electrospun Short Fibrous Sponge via Engineered Nanofat for Wound Healing,” Advanced Fiber Materials 5 (2023): 979–993.

[26] V. Di Nicola , “Omentum a Powerful Biological Source in Regenerative Surgery,” Regenerative Therapy 11 (2019): 182–191. 31453273 10.1016/j.reth.2019.07.008 PMC6700267

[27] C. W. Ha , A. Martin , G. D. Sepich‐Poore , et al., “Translocation of Viable Gut Microbiota to Mesenteric Adipose Drives Formation of Creeping Fat in Humans,” Cell 183 (2020): 666–683. 32991841 10.1016/j.cell.2020.09.009 PMC7521382

[28] G. Wang , A. Song , and Q. A. Wang , “Adipose Tissue Ageing: Implications for Metabolic Health and Lifespan,” Nature Reviews Endocrinology 21 (2025): 623–637. 10.1038/s41574-025-01142-8 40550948

[29] J. W. Porter , J. L. Barnas , R. Welly , et al., “Age, Sex, and Depot‐Specific Differences in Adipose‐Tissue Estrogen Receptors in Individuals With Obesity,” Obesity 28 (2020): 1698–1707. 32734695 10.1002/oby.22888 PMC7483923

[30] J. He , F. Chen , Y. Zhang , P. Tan , Q. Li , and C. Cheng , “Concentrated Ultrasound‐Processed Fat (CUPF): More Than a Mechanically Emulsified Graft,” Journal of Plastic, Reconstructive & Aesthetic Surgery 83 (2023): 198–206. 10.1016/j.bjps.2023.04.073 37279632

[31] F. Xie , Y. Liu , H. Wang , Y. Zhang , and Y. Xie , “Association Between Liposuction and Menstrual Cycle Changes: A Retrospective Study,” Plastic and Aesthetic Research 12 (2025): 15.

[32] Z. Li , K. Xu , S. Zhao , et al., “SPATA4 Improves Aging‐Induced Metabolic Dysfunction Through Promotion of Preadipocyte Differentiation and Adipose Tissue Expansion,” Aging Cell 20 (2021): e13282. 33314576 10.1111/acel.13282 PMC7811838

[33] R. B. S. Turner , D. Tyrrell , G. Hepworth , F. R. Dunshea , and C. S. Mansfield , “Compartmental Fat Distribution in the Abdomen of Dogs Relative to Overall Body Fat Composition,” BMC Veterinary Research 16 (2020): 104. 32228685 10.1186/s12917-020-02327-1 PMC7106746

[34] B. A. McKenzie , “Comparative Veterinary Geroscience: Mechanism of Molecular, Cellular, and Tissue Aging in Humans, Laboratory Animal Models, and Companion Dogs and Cats,” American Journal of Veterinary Research 83 (2022): ajvr.22.02.0027. 35524953 10.2460/ajvr.22.02.0027

[35] A. Mansoor , L. Salciccioli , G. Qureshi , et al., “Echocardiographic Determination of Epicardial Adipose Tissue in Healthy Bonnet Macaques,” Echocardiography 27 (2010): 180–185. 19725843 10.1111/j.1540-8175.2009.00991.x

[36] Y. Yan , H. Wang , M. Hu , et al., “HDAC6 Suppresses Age‐Dependent Ectopic Fat Accumulation by Maintaining the Proteostasis of PLIN2 in Drosophila,” Developmental Cell 43 (2017): 99–111. 28966044 10.1016/j.devcel.2017.09.001

[37] Y. Zhang , J. He , F. Xie , et al., “Spatial Transcriptomic Analysis Deciphers Adipocyte‐to‐Fibroblast Transformation in Bleomycin‐Induced Murine Skin Fibrosis,” Chinese Medical Journal 137 (2024): 2745–2757. 39345020 10.1097/CM9.0000000000003219 PMC11611235

[38] T. Sasaki , Y. Nishimoto , T. Hirata , et al., “Status and Physiological Significance of Circulating Adiponectin in the Very Old and Centenarians: An Observational Study,” eLife 12 (2023): e86309. 37768324 10.7554/eLife.86309 PMC10564453

[39] N. Li , S. Zhao , Z. Zhang , et al., “Adiponectin Preserves Metabolic Fitness During Aging,” eLife 10 (2021): e65108. 33904399 10.7554/eLife.65108 PMC8099426

[40] S. Khan , Y. T. Chan , X. S. Revelo , and D. A. Winer , “The Immune Landscape of Visceral Adipose Tissue During Obesity and Aging,” Frontiers in Endocrinology 11 (2020): 267. 32499756 10.3389/fendo.2020.00267 PMC7243349

[41] Y. Zhang , B. Fang , and Y. Xie , “A Novel Composite Skin Graft Technique With Fat Derivatives,” Chinese Journal of Plastic and Reconstructive Surgery 3 (2021): 82–84.

[42] C. N. Lumeng , J. Liu , L. Geletka , et al., “Aging Is Associated With an Increase in T Cells and Inflammatory Macrophages in Visceral Adipose Tissue,” Journal of Immunology 187 (2011): 6208–6216. 10.4049/jimmunol.1102188 PMC3237772 22075699

[43] A. Gavaldà‐Navarro , J. Villarroya , R. Cereijo , M. Giralt , and F. Villarroya , “The Endocrine Role of Brown Adipose Tissue: An Update on Actors and Actions,” Reviews in Endocrine & Metabolic Disorders 23 (2022): 31–41. 33712997 10.1007/s11154-021-09640-6

[44] L. Massier , J. Jalkanen , M. Elmastas , et al., “An Integrated Single Cell and Spatial Transcriptomic Map of Human White Adipose Tissue,” Nature Communications 14 (2023): 1438. 10.1038/s41467-023-36983-2 PMC10017705 36922516

[45] B. Maniyadath , Q. Zhang , R. K. Gupta , and S. Mandrup , “Adipose Tissue at Single‐Cell Resolution,” Cell Metabolism 35 (2023): 386–413. 36889280 10.1016/j.cmet.2023.02.002 PMC10027403

[46] S. Corvera , “Cellular Heterogeneity in Adipose Tissues,” Annual Review of Physiology 83 (2021): 257–278. 10.1146/annurev-physiol-031620-095446 PMC8091658 33566675

[47] G. Matacchione , J. Perugini , E. Di Mercurio , et al., “Senescent Macrophages in the Human Adipose Tissue as a Source of Inflammaging,” GeroScience 44 (2022): 1941–1960. 35247131 10.1007/s11357-022-00536-0 PMC9616990

[48] C. Franceschi , M. Bonafè , S. Valensin , et al., “Inflamm‐Aging: An Evolutionary Perspective on Immunosenescence,” Annals of the New York Academy of Sciences 908 (2000): 244–254. 10911963 10.1111/j.1749-6632.2000.tb06651.x

[49] Z. Yang Loureiro , S. Joyce , T. DeSouza , et al., “Wnt Signaling Preserves Progenitor Cell Multipotency During Adipose Tissue Development,” Nature Metabolism 5 (2023): 1014–1028. 10.1038/s42255-023-00813-y PMC10290956 37337125

[50] E. Arner , P. O. Westermark , K. L. Spalding , et al., “Adipocyte Turnover: Relevance to Human Adipose Tissue Morphology,” Diabetes 59 (2010): 105–109. 19846802 10.2337/db09-0942 PMC2797910

[51] R. Z. Ye , E. Montastier , F. Frisch , et al., “Adipocyte Hypertrophy Associates With In Vivo Postprandial Fatty Acid Metabolism and Adipose Single‐Cell Transcriptional Dynamics,” iScience 27 (2024): 108692. 38226167 10.1016/j.isci.2023.108692 PMC10788217

[52] F. Liu , J. He , H. Wang , D. Zhu , and Y. Bi , “Adipose Morphology: A Critical Factor in Regulation of Human Metabolic Diseases and Adipose Tissue Dysfunction,” Obesity Surgery 30 (2020): 5086–5100. 33021706 10.1007/s11695-020-04983-6 PMC7719100

[53] B. Craig , S. Garthwaite , and J. Holloszy , “Adipocyte Insulin Resistance: Effects of Aging, Obesity, Exercise, and Food Restriction,” Journal of Applied Physiology 62 (1987): 95–100. 3549672 10.1152/jappl.1987.62.1.95

[54] K. Verboven , K. Wouters , K. Gaens , et al., “Abdominal Subcutaneous and Visceral Adipocyte Size, Lipolysis and Inflammation Relate to Insulin Resistance in Male Obese Humans,” Scientific Reports 8 (2018): 4677. 29549282 10.1038/s41598-018-22962-x PMC5856747

[55] C. D. Camell , P. Günther , A. Lee , et al., “Aging Induces an Nlrp3 Inflammasome‐Dependent Expansion of Adipose B Cells That Impairs Metabolic Homeostasis,” Cell Metabolism 30 (2019): 1024–1039. 31735593 10.1016/j.cmet.2019.10.006 PMC6944439

[56] S. P. Bapat , J. Myoung Suh , S. Fang , et al., “Depletion of Fat‐Resident Treg Cells Prevents Age‐Associated Insulin Resistance,” Nature 528 (2015): 137–141. 26580014 10.1038/nature16151 PMC4670283

[57] D. Brigger , C. Riether , R. van Brummelen , et al., “Eosinophils Regulate Adipose Tissue Inflammation and Sustain Physical and Immunological Fitness in Old Age,” Nature Metabolism 2 (2020): 688–702. 10.1038/s42255-020-0228-3 PMC7438316 32694825

[58] S. Corvera , J. Solivan‐Rivera , and Z. Yang Loureiro , “Angiogenesis in Adipose Tissue and Obesity,” Angiogenesis 25 (2022): 439–453. 35857195 10.1007/s10456-022-09848-3 PMC9519636

[59] J. Honek , T. Seki , H. Iwamoto , et al., “Modulation of Age‐Related Insulin Sensitivity by VEGF‐Dependent Vascular Plasticity in Adipose Tissues,” Proceedings of the National Academy of Sciences 111 (2014): 14906–14911. 10.1073/pnas.1415825111 PMC4205611 25271320

[60] S. M. Kim , M. Lun , M. Wang , et al., “Loss of White Adipose Hyperplastic Potential Is Associated With Enhanced Susceptibility to Insulin Resistance,” Cell Metabolism 20 (2014): 1049–1058. 25456741 10.1016/j.cmet.2014.10.010 PMC4715375

[61] Y. Lin , X. Fan , L. Tang , Y. Tang , and J. Gu , “Polysilsesquioxane‐PBO Wave‐Transparent Composite Paper With Excellent Mechanical Properties and Ultraviolet Aging Resistance,” Advanced Fiber Materials 5 (2023): 2114–2126.

[62] J. Chen , S. Ren , Z. Chen , et al., “Plasticity Enhancement in Metallic Glasses via Aging‐Assisted Ultrasonic Vibrations,” Materials Futures 4 (2025): 035003.

[63] J. L. Kirkland , C. H. Hollenberg , S. Kindler , and W. S. Gillon , “Effects of Age and Anatomic Site on Preadipocyte Number in Rat Fat Depots,” Journal of Gerontology 49 (1994): B31–B35. 8282974 10.1093/geronj/49.1.b31

[64] H. T. Chen , M. Lee , C. Chen , et al., “Proliferation and Differentiation Potential of Human Adipose‐Derived Mesenchymal Stem Cells Isolated From Elderly Patients With Osteoporotic Fractures,” Journal of Cellular and Molecular Medicine 16 (2012): 582–592. 21545685 10.1111/j.1582-4934.2011.01335.x PMC3822933

[65] E. U. Alt , C. Senst , S. N. Murthy , et al., “Aging Alters Tissue Resident Mesenchymal Stem Cell Properties,” Stem Cell Research 8 (2012): 215–225. 22265741 10.1016/j.scr.2011.11.002

[66] H. Jiang , Z. Lin , J. Li , et al., “rADSC‐Loaded Tubular Units Composed of Multilayer Electrospun Membranes Promoted Bone Regeneration of Critical‐Sized Skull Defects,” Materials Futures 3 (2024): 035403.

[67] M. Zhu , E. Kohan , J. Bradley , M. Hedrick , P. Benhaim , and P. Zuk , “The Effect of Age on Osteogenic, Adipogenic and Proliferative Potential of Female Adipose‐Derived Stem Cells,” Journal of Tissue Engineering and Regenerative Medicine 3 (2009): 290–301. 19309766 10.1002/term.165

[68] I. Karagiannides , T. Tchkonia , D. E. Dobson , et al., “Altered Expression of C/EBP Family Members Results in Decreased Adipogenesis With Aging,” American Journal of Physiology – Regulatory, Integrative and Comparative Physiology 280 (2001): R1772–R1780. 11353682 10.1152/ajpregu.2001.280.6.R1772

[69] K. Hotta , N. L. Bodkin , T. A. Gustafson , S. Yoshioka , H. K. Ortmeyer , and B. C. Hansen , “Age‐Related Adipose Tissue mRNA Expression of ADD1/SREBP1, PPARγ, Lipoprotein Lipase, and GLUT4 Glucose Transporter in Rhesus Monkeys,” Journals of Gerontology, Series A: Biological Sciences and Medical Sciences 54 (1999): B183–B188. 10361996 10.1093/gerona/54.5.b183

[70] I. Karagiannides , T. Thomou , T. Tchkonia , et al., “Increased CUG Triplet Repeat‐Binding Protein‐1 Predisposes to Impaired Adipogenesis With Aging,” Journal of Biological Chemistry 281 (2006): 23025–23033. 16754681 10.1074/jbc.M513187200

[71] T. Tchkonia , T. Pirtskhalava , T. Thomou , et al., “Increased TNFα and CCAAT/Enhancer‐Binding Protein Homologous Protein With Aging Predispose Preadipocytes to Resist Adipogenesis,” American Journal of Physiology‐Endocrinology and Metabolism 293 (2007): E1810–E1819. 17911345 10.1152/ajpendo.00295.2007

[72] J. Fei , H. Tamski , C. Cook , and N. Santanam , “MicroRNA Regulation of Adipose Derived Stem Cells in Aging Rats,” PLoS One 8 (2013): e59238. 23516615 10.1371/journal.pone.0059238 PMC3597632

[73] M. Xu , A. K. Palmer , H. Ding , et al., “Targeting Senescent Cells Enhances Adipogenesis and Metabolic Function in Old Age,” eLife 4 (2015): e12997. 26687007 10.7554/eLife.12997 PMC4758946

[74] M. C. Mitterberger , S. Lechner , M. Mattesich , and W. Zwerschke , “Adipogenic Differentiation Is Impaired in Replicative Senescent Human Subcutaneous Adipose‐Derived Stromal/Progenitor Cells,” Journals of Gerontology, Series A: Biological Sciences and Medical Sciences 69 (2014): 13–24. 23657974 10.1093/gerona/glt043

[75] S. M. Conley , L. J. Hickson , T. A. Kellogg , et al., “Human Obesity Induces Dysfunction and Early Senescence in Adipose Tissue‐Derived Mesenchymal Stromal/Stem Cells,” Frontiers in Cell and Developmental Biology 8 (2020): 197. 32274385 10.3389/fcell.2020.00197 PMC7113401

[76] S. Pauklin and L. Vallier , “The Cell‐Cycle State of Stem Cells Determines Cell Fate Propensity,” Cell 155 (2013): 135–147. 24074866 10.1016/j.cell.2013.08.031 PMC3898746

[77] X. Shan and I. Percec , “Mechanisms of Adipose‐Derived Stem Cell Aging and the Impact on Therapeutic Potential,” in Scientific Principles of Adipose Stem Cells, ed. L. Kokai , K. Marra , and J. P. Rubin (Academic Press, 2021), 81–90.

[78] J. Oh , Y. D. Lee , and A. J. Wagers , “Stem Cell Aging: Mechanisms, Regulators and Therapeutic Opportunities,” Nature Medicine 20 (2014): 870–880. 10.1038/nm.3651 PMC4160113 25100532

[79] M. Liu , H. Lei , P. Dong , et al., “Adipose‐Derived Mesenchymal Stem Cells From the Elderly Exhibit Decreased Migration and Differentiation Abilities With Senescent Properties,” Cell Transplantation 26 (2017): 1505–1519. 29113467 10.1177/0963689717721221 PMC5680952

[80] M. C. Haigis and B. A. Yankner , “The Aging Stress Response,” Molecular Cell 40 (2010): 333–344. 20965426 10.1016/j.molcel.2010.10.002 PMC2987618

[81] D. J. Dues , E. K. Andrews , C. E. Schaar , A. L. Bergsma , M. M. Senchuk , and J. M. Van Raamsdonk , “Aging Causes Decreased Resistance to Multiple Stresses and a Failure to Activate Specific Stress Response Pathways,” Aging (Albany NY) 8 (2016): 777–795. 27053445 10.18632/aging.100939 PMC4925828

[82] S. Heinonen , R. Jokinen , A. Rissanen , and K. H. Pietiläinen , “White Adipose Tissue Mitochondrial Metabolism in Health and in Obesity,” Obesity Reviews 21 (2020): e12958. 31777187 10.1111/obr.12958

[83] J. A. Amorim , G. Coppotelli , A. P. Rolo , C. M. Palmeira , J. M. Ross , and D. A. Sinclair , “Mitochondrial and Metabolic Dysfunction in Ageing and Age‐Related Diseases,” Nature Reviews Endocrinology 18 (2022): 243–258. 10.1038/s41574-021-00626-7 PMC9059418 35145250

[84] A. H. de Mello , A. B. Costa , J. D. G. Engel , and G. T. Rezin , “Mitochondrial Dysfunction in Obesity,” Life Sciences 192 (2018): 26–32. 29155300 10.1016/j.lfs.2017.11.019

[85] C. Mas‐Bargues , “Mitochondria Pleiotropism in Stem Cell Senescence: Mechanisms and Therapeutic Approaches,” Free Radical Biology and Medicine 208 (2023): 657–671. 37739140 10.1016/j.freeradbiomed.2023.09.019

[86] A. Bratic and N. G. Larsson , “The Role of Mitochondria in Aging,” Journal of Clinical Investigation 123 (2013): 951–957. 23454757 10.1172/JCI64125 PMC3582127

[87] Y. Zhang , H. Yang , D. Wei , et al., “Mitochondria‐Targeted Nanoparticles in Treatment of Neurodegenerative Diseases,” Exploration 1 (2021): 20210115. 37323688 10.1002/EXP.20210115 PMC10191038

[88] D. Harman , “Aging: A Theory Based on Free Radical and Radiation Chemistry,” Journal of Gerontology 11 (1956): 298–300. 13332224 10.1093/geronj/11.3.298

[89] N. G. Larsson , “Somatic Mitochondrial DNA Mutations in Mammalian Aging,” Annual Review of Biochemistry 79 (2010): 683–706. 10.1146/annurev-biochem-060408-093701 20350166

[90] S. Victorelli , H. Salmonowicz , J. Chapman , et al., “Apoptotic Stress Causes mtDNA Release During Senescence and Drives the SASP,” Nature 622 (2023): 627–636. 37821702 10.1038/s41586-023-06621-4 PMC10584674

[91] J. Yang , S. Wu , and M. He , “Engineered Exosome‐Based Senolytic Therapy Alleviates Stroke by Targeting p21^+^CD86^+^ Microglia,” Exploration 5 (2025): 20240349. 40585759 10.1002/EXP.20240349 PMC12199405

[92] Y. Wan and T. Finkel , “The Mitochondria Regulation of Stem Cell Aging,” Mechanisms of Ageing and Development 191 (2020): 111334. 32818514 10.1016/j.mad.2020.111334 PMC7541753

[93] J. Yu , Y. Huang , Y. Qin , et al., “Deciphering Mitochondria: Unveiling Their Roles in Mechanosensing and Mechanotransduction,” Research 8 (2025): 0816. 40785969 10.34133/research.0816 PMC12332263

[94] S. Miwa , S. Kashyap , E. Chini , and T. von Zglinicki , “Mitochondrial Dysfunction in Cell Senescence and Aging,” Journal of Clinical Investigation 132 (2022): e158447. 35775483 10.1172/JCI158447 PMC9246372

[95] W. Zhang , H. Xiong , J. Pang , et al., “PGC‐1α Overexpression Promotes Mitochondrial Biogenesis to Protect Auditory Cells Against Cisplatin‐Induced Cytotoxicity,” Journal of Bio‐X Research 2 (2019): 81–86.

[96] I. A. Khan , T. Yu , M. Yang , J. Liu , and Z. Chen , “A Systematic Review of Toxicity, Biodistribution, and Biosafety in Upconversion Nanomaterials: Critical Insights Into Toxicity Mitigation Strategies and Future Directions for Safe Applications,” BME Frontiers 6 (2025): 0120. 40416504 10.34133/bmef.0120 PMC12099058

[97] A. Trifunovic , A. Wredenberg , M. Falkenberg , et al., “Premature Ageing in Mice Expressing Defective Mitochondrial DNA Polymerase,” Nature 429 (2004): 417–423. 15164064 10.1038/nature02517

[98] K. J. Ahlqvist , R. H. Hämäläinen , S. Yatsuga , et al., “Somatic Progenitor Cell Vulnerability to Mitochondrial DNA Mutagenesis Underlies Progeroid Phenotypes in Polg Mutator Mice,” Cell Metabolism 15 (2012): 100–109. 22225879 10.1016/j.cmet.2011.11.012

[99] B. Spurlock , J. Tullet , J. L. Hartman IV , and K. Mitra , “Interplay of Mitochondrial Fission‐Fusion With Cell Cycle Regulation: Possible Impacts on Stem Cell and Organismal Aging,” Experimental Gerontology 135 (2020): 110919. 32220593 10.1016/j.exger.2020.110919 PMC7808294

[100] J. Zhou , S. Jiang , L. Wang , et al., “Decellularized Adipose Matrix Rejuvenates Photoaged Skin Through Immune Microenvironment Modulation,” BME Frontiers 6 (2025): 0166. 40761470 10.34133/bmef.0166 PMC12320489

[101] Y. Wang , X. Ma , W. Zhou , C. Liu , and H. Zhang , “Reregulated Mitochondrial Dysfunction Reverses Cisplatin Resistance Microenvironment in Colorectal Cancer,” Smart Medicine 1 (2022): e20220013. 39188744 10.1002/SMMD.20220013 PMC11235731

[102] H. Martini and J. F. Passos , “Cellular Senescence: All Roads Lead to Mitochondria,” FEBS Journal 290 (2023): 1186–1202. 35048548 10.1111/febs.16361 PMC9296701

[103] Y. S. Yoon , D. Yoon , I. K. Lim , et al., “Formation of Elongated Giant Mitochondria in DFO‐Induced Cellular Senescence: Involvement of Enhanced Fusion Process Through Modulation of Fis1,” Journal of Cellular Physiology 209 (2006): 468–480. 16883569 10.1002/jcp.20753

[104] P. P. Naik , A. Birbrair , and S. K. Bhutia , “Mitophagy‐Driven Metabolic Switch Reprograms Stem Cell Fate,” Cellular and Molecular Life Sciences 76 (2019): 27–43. 30267101 10.1007/s00018-018-2922-9 PMC11105479

[105] C. Correia‐Melo , F. D. Marques , R. Anderson , et al., “Mitochondria Are Required for Pro‐Ageing Features of the Senescent Phenotype,” EMBO Journal 35 (2016): 724–742. 26848154 10.15252/embj.201592862 PMC4818766

[106] W. Chen , M. Nie , J. Gan , N. Xia , D. Wang , and L. Sun , “Tailoring Cell Sheets for Biomedical Applications,” Smart Medicine 3 (2024): e20230038. 39188516 10.1002/SMMD.20230038 PMC11235941

[107] X. Xu , S. Duan , F. Yi , A. Ocampo , G. H. Liu , and J. C. I. Belmonte , “Mitochondrial Regulation in Pluripotent Stem Cells,” Cell Metabolism 18 (2013): 325–332. 23850316 10.1016/j.cmet.2013.06.005

[108] H. Han , J. Li , and H. A. Santos , “Recent Advances in Fenton and Fenton‐Like Reaction Mediated Nanoparticle in Cancer Therapy,” Biomedical Technology 3 (2023): 40–51.

[109] E. Hutter , K. Renner , G. Pfister , P. Stöckl , P. Jansen‐Dürr , and E. Gnaiger , “Senescence‐Associated Changes in Respiration and Oxidative Phosphorylation in Primary Human Fibroblasts,” Biochemical Journal 380 (2004): 919–928. 15018610 10.1042/BJ20040095 PMC1224220

[110] J. Zhang , Y. Yan , J. Wan , Y. Zhang , and J. Zhou , “Gαi1 Activation Induced by Short‐Term Hypoxia Promotes Epidermal Cell Migration in Wound Healing Through the Akt‐mTOR Pathway,” Biomedical Technology 10 (2025): 100072.

[111] R. G. Jones , D. R. Plas , S. Kubek , et al., “AMP‐Activated Protein Kinase Induces a p53‐Dependent Metabolic Checkpoint,” Molecular Cell 18 (2005): 283–293. 15866171 10.1016/j.molcel.2005.03.027

[112] P. Jiang , W. Du , A. Mancuso , K. E. Wellen , and X. Yang , “Reciprocal Regulation of p53 and Malic Enzymes Modulates Metabolism and Senescence,” Nature 493 (2013): 689–693. 23334421 10.1038/nature11776 PMC3561500

[113] I. Ben‐Porath and R. A. Weinberg , “The Signals and Pathways Activating Cellular Senescence,” International Journal of Biochemistry & Cell Biology 37 (2005): 961–976. 15743671 10.1016/j.biocel.2004.10.013

[114] L. Jia , Z. Wei , J. Luoqian , X. Wang , and C. Huang , “Mitochondrial Dysfunction in Aging: Future Therapies and Precision Medicine Approaches,” MedComm – Future Medicine 4 (2025): e70026.

[115] M. Xia , Y. Zhang , K. Jin , Z. Lu , Z. Zeng , and W. Xiong , “Communication Between Mitochondria and Other Organelles: A Brand‐New Perspective on Mitochondria in Cancer,” Cell & Bioscience 9 (2019): 27. 30931098 10.1186/s13578-019-0289-8 PMC6425566

[116] D. Zhu , X. Li , and Y. Tian , “Mitochondrial‐to‐Nuclear Communication in Aging: An Epigenetic Perspective,” Trends in Biochemical Sciences 47 (2022): 645–659. 35397926 10.1016/j.tibs.2022.03.008

[117] P. M. Quirós , A. Mottis , and J. Auwerx , “Mitonuclear Communication in Homeostasis and Stress,” Nature Reviews Molecular Cell Biology 17 (2016): 213–226. 26956194 10.1038/nrm.2016.23

[118] E. F. Fang , M. Scheibye‐Knudsen , K. F. Chua , M. P. Mattson , D. L. Croteau , and V. A. Bohr , “Nuclear DNA Damage Signalling to Mitochondria in Ageing,” Nature Reviews Molecular Cell Biology 17 (2016): 308–321. 26956196 10.1038/nrm.2016.14 PMC5161407

[119] O. Matilainen , P. M. Quirós , and J. Auwerx , “Mitochondria and Epigenetics – Crosstalk in Homeostasis and Stress,” Trends in Cell Biology 27 (2017): 453–463. 28274652 10.1016/j.tcb.2017.02.004

[120] T. R. Mercer , S. Neph , M. Dinger , et al., “The Human Mitochondrial Transcriptome,” Cell 146 (2011): 645–658. 21854988 10.1016/j.cell.2011.06.051 PMC3160626

[121] S. S. Dhar , S. Ongwijitwat , and M. T. T. Wong‐Riley , “Nuclear Respiratory Factor 1 Regulates All Ten Nuclear‐Encoded Subunits of Cytochrome C Oxidase in Neurons,” Journal of Biological Chemistry 283 (2008): 3120–3129. 18077450 10.1074/jbc.M707587200 PMC2669777

[122] J. V. Virbasius and R. C. Scarpulla , “Activation of the Human Mitochondrial Transcription Factor A Gene by Nuclear Respiratory Factors: A Potential Regulatory Link Between Nuclear and Mitochondrial Gene Expression in Organelle Biogenesis,” Proceedings of the National Academy of Sciences of the United States of America 91 (1994): 1309–1313. 8108407 10.1073/pnas.91.4.1309 PMC43147

[123] T. Tanaka , J. Yamamoto , S. Iwasaki , et al., “Activation of Peroxisome Proliferator‐Activated Receptor δ Induces Fatty Acid β‐Oxidation in Skeletal Muscle and Attenuates Metabolic Syndrome,” Proceedings of the National Academy of Sciences of the United States of America 100 (2003): 15924–15929. 14676330 10.1073/pnas.0306981100 PMC307669

[124] C. R. Dufour , B. J. Wilson , J. M. Huss , et al., “Genome‐Wide Orchestration of Cardiac Functions by the Orphan Nuclear Receptors ERRα and γ,” Cell Metabolism 5 (2007): 345–356. 17488637 10.1016/j.cmet.2007.03.007

[125] S. Peralta , X. Wang , and C. T. Moraes , “Mitochondrial Transcription: Lessons From Mouse Models,” Biochimica et Biophysica Acta (BBA)‐Gene Regulatory Mechanisms 1819 (2012): 961–969. 22120174 10.1016/j.bbagrm.2011.11.001 PMC3408808

[126] X. Luo and W. L. Kraus , “On PAR With PARP: Cellular Stress Signaling Through Poly(ADP‐Ribose) and PARP‐1,” Genes & Development 26 (2012): 417–432. 22391446 10.1101/gad.183509.111 PMC3305980

[127] P. Bai , C. Cantó , H. Oudart , et al., “PARP‐1 Inhibition Increases Mitochondrial Metabolism Through SIRT1 Activation,” Cell Metabolism 13 (2011): 461–468. 21459330 10.1016/j.cmet.2011.03.004 PMC3086520

[128] J. Rosen , P. Jakobs , N. Ale‐Agha , J. Altschmied , and J. Haendeler , “Non‐Canonical Functions of Telomerase Reverse Transcriptase – Impact on Redox Homeostasis,” Redox Biology 34 (2020): 101543. 32502898 10.1016/j.redox.2020.101543 PMC7267725

[129] W. Chen , Z. Zhao , Z. Geng , H. Zhang , and X. Fu , “Advances in Mitochondria‐Nucleus Crosstalk in Septic Cardiomyopathy,” Cell Biology and Toxicology 41 (2025): 136. 41051583 10.1007/s10565-025-10090-y PMC12500838

[130] G. Darfarin and J. Pluth , “Mitochondria‐Nuclear Crosstalk: Orchestrating mtDNA Maintenance,” Environmental and Molecular Mutagenesis 66 (2025): 222–242. 40418056 10.1002/em.70013 PMC12235075

[131] B. Peng , Y. Wang , and H. Zhang , “Mitonuclear Communication in Stem Cell Function,” Cell Proliferation 58 (2025): e13796. 39726221 10.1111/cpr.13796 PMC12099226

[132] R. M. Chin , X. Fu , M. Y. Pai , et al., “The Metabolite α‐Ketoglutarate Extends Lifespan by Inhibiting ATP Synthase and TOR,” Nature 510 (2014): 397–401. 24828042 10.1038/nature13264 PMC4263271

[133] R. M. N. Friis , J. P. Glaves , T. Huan , L. Li , B. D. Sykes , and M. C. Schultz , “Rewiring AMPK and Mitochondrial Retrograde Signaling for Metabolic Control of Aging and Histone Acetylation in Respiratory‐Defective Cells,” Cell Reports 7 (2014): 565–574. 24726357 10.1016/j.celrep.2014.03.029

[134] Y. Ozaki , K. Ohashi , N. Otaka , et al., “Myonectin Protects Against Skeletal Muscle Dysfunction in Male Mice Through Activation of AMPK/PGC1α Pathway,” Nature Communications 14 (2023): 4675. 10.1038/s41467-023-40435-2 PMC10403505 37542026

[135] S. Herzig and R. J. Shaw , “AMPK: Guardian of Metabolism and Mitochondrial Homeostasis,” Nature Reviews Molecular Cell Biology 19 (2018): 121–135. 28974774 10.1038/nrm.2017.95 PMC5780224

[136] C. Lerner , A. Bitto , D. Pulliam , et al., “Reduced Mammalian Target of Rapamycin Activity Facilitates Mitochondrial Retrograde Signaling and Increases Life Span in Normal Human Fibroblasts,” Aging Cell 12 (2013): 966–977. 23795962 10.1111/acel.12122 PMC5559196

[137] S. Patergnani , J. M. Suski , C. Agnoletto , et al., “Calcium Signaling Around Mitochondria Associated Membranes (MAMs),” Cell Communication and Signaling 9 (2011): 19. 21939514 10.1186/1478-811X-9-19 PMC3198985

[138] L. Formentini , M. Sánchez‐Aragó , L. Sánchez‐Cenizo , and J. M. Cuezva , “The Mitochondrial ATPase Inhibitory Factor 1 Triggers a ROS‐Mediated Retrograde Prosurvival and Proliferative Response,” Molecular Cell 45 (2012): 731–742. 22342343 10.1016/j.molcel.2012.01.008

[139] S. Chae , B. Y. Ahn , K. Byun , et al., “A Systems Approach for Decoding Mitochondrial Retrograde Signaling Pathways,” Science Signaling 6 (2013): rs4. 23443683 10.1126/scisignal.2003266

[140] R. Acín‐Pérez , I. Carrascoso , F. Baixauli , et al., “ROS‐Triggered Phosphorylation of Complex II by Fgr Kinase Regulates Cellular Adaptation to Fuel Use,” Cell Metabolism 19 (2014): 1020–1033. 24856931 10.1016/j.cmet.2014.04.015 PMC4274740

[141] S. Victorelli , M. Eppard , H. Martini , et al., “Mitochondrial RNA Cytosolic Leakage Drives the SASP,” Nature Communications 16 (2025): 10992. 10.1038/s41467-025-66159-z PMC12705736 41398033

[142] S. Mishra , N. Welch , M. Karthikeyan , et al., “Dysregulated Cellular Redox Status During Hyperammonemia Causes Mitochondrial Dysfunction and Senescence by Inhibiting Sirtuin‐Mediated Deacetylation,” Aging Cell 22 (2023): e13852. 37101412 10.1111/acel.13852 PMC10352558

[143] O. F. Kuzu , L. J. T. Granerud , and F. Saatcioglu , “Navigating the Landscape of Protein Folding and Proteostasis: From Molecular Chaperones to Therapeutic Innovations,” Signal Transduction and Targeted Therapy 10 (2025): 358. 41130962 10.1038/s41392-025-02439-w PMC12550075

[144] V. Jovaisaite , L. Mouchiroud , and J. Auwerx , “The Mitochondrial Unfolded Protein Response, a Conserved Stress Response Pathway With Implications in Health and Disease,” Journal of Experimental Biology 217 (2014): 137–143. 24353213 10.1242/jeb.090738 PMC3867496

[145] M. Picard and O. S. Shirihai , “Mitochondrial Signal Transduction,” Cell Metabolism 34 (2022): 1620–1653. 36323233 10.1016/j.cmet.2022.10.008 PMC9692202

[146] J. Jena , L. M. García‐Peña , and R. O. Pereira , “The Roles of FGF21 and GDF15 in Mediating the Mitochondrial Integrated Stress Response,” Frontiers in Endocrinology 14 (2023): 1264530. 37818094 10.3389/fendo.2023.1264530 PMC10561105

[147] N. R. S. Sibuyi , K. L. Moabelo , M. Meyer , M. O. Onani , A. Dube , and A. M. Madiehe , “Nanotechnology Advances Towards Development of Targeted‐Treatment for Obesity,” Journal of Nanobiotechnology 17 (2019): 122. 31842876 10.1186/s12951-019-0554-3 PMC6913004

[148] D. Zhao , Y. Zhang , F. Wang , et al., “Drug‐Phospholipid Conjugate Nano‐Assembly for Drug Delivery,” Smart Medicine 3 (2024): e20240053. 39776594 10.1002/SMMD.20240053 PMC11669785

[149] M. G. Kolonin , P. K. Saha , L. Chan , R. Pasqualini , and W. Arap , “Reversal of Obesity by Targeted Ablation of Adipose Tissue,” Nature Medicine 10 (2004): 625–632. 10.1038/nm1048 15133506

[150] R. Hiradate , I. A. Khalil , A. Matsuda , M. Sasaki , K. Hida , and H. Harashima , “A Novel Dual‐Targeted Rosiglitazone‐Loaded Nanoparticle for the Prevention of Diet‐Induced Obesity via the Browning of White Adipose Tissue,” Journal of Controlled Release 329 (2021): 665–675. 33038450 10.1016/j.jconrel.2020.10.002

[151] B. Yu , Y. Pu , J. Liu , et al., “Targeted Delivery of Emodin to Adipocytes by Aptamer‐Functionalized PEG‐PLGA Nanoparticles In Vitro,” Journal of Drug Delivery Science and Technology 57 (2020): 101739.

[152] B. Huang , Q. Wan , T. Li , et al., “Polycationic PAMAM Ameliorates Obesity‐Associated Chronic Inflammation and Focal Adiposity,” Biomaterials 293 (2023): 121850. 36450630 10.1016/j.biomaterials.2022.121850 PMC12433583

[153] Q. Wan , B. Huang , T. Li , et al., “Selective Targeting of Visceral Adiposity by Polycation Nanomedicine,” Nature Nanotechnology 17 (2022): 1311–1321. 10.1038/s41565-022-01249-3 36456644

[154] F. Abedi‐Gaballu , G. Dehghan , M. Ghaffari , et al., “PAMAM Dendrimers as Efficient Drug and Gene Delivery Nanosystems for Cancer Therapy,” Applied Materials Today 12 (2018): 177–190. 30511014 10.1016/j.apmt.2018.05.002 PMC6269116

[155] A. C. Daquinag , A. Dadbin , B. Snyder , et al., “Non‐Glycanated Decorin Is a Drug Target on Human Adipose Stromal Cells,” Molecular Therapy Oncolytics 6 (2017): 1–9. 28607949 10.1016/j.omto.2017.05.003 PMC5458115

[156] S. Ling , Y. Zhang , Y. Chen , et al., “Ionizable Coenzyme‐Engineered Lipid/Fiber Microplexes Boost Ribosomal Translation to Improve mRNA Therapy for Degenerative Diseases,” Advanced Materials 38 (2026): e13720. 41058519 10.1002/adma.202513720

[157] J. Nandakumar and T. R. Cech , “Finding the End: Recruitment of Telomerase to Telomeres,” Nature Reviews Molecular Cell Biology 14 (2013): 69–82. 23299958 10.1038/nrm3505 PMC3805138

[158] K. Okuda , A. Bardeguez , J. P. Gardner , et al., “Telomere Length in the Newborn,” Pediatric Research 52 (2002): 377–381. 12193671 10.1203/00006450-200209000-00012

[159] K. A. Lewis and D. S. Wuttke , “Telomerase and Telomere‐Associated Proteins: Structural Insights Into Mechanism and Evolution,” Structure 20 (2012): 28–39. 22244753 10.1016/j.str.2011.10.017 PMC4180718

[160] Y. J. Chiang , M. T. Hemann , K. S. Hathcock , et al., “Expression of Telomerase RNA Template, but Not Telomerase Reverse Transcriptase, Is Limiting for Telomere Length Maintenance In Vivo,” Molecular and Cellular Biology 24 (2004): 7024–7031. 15282303 10.1128/MCB.24.16.7024-7031.2004 PMC479722

[161] D. S. Nalobin , A. A. Galiakberova , S. I. Alipkina , and A. I. Glukhov , “Regulation of Telomerase Activity,” Biology Bulletin Reviews 8 (2018): 142–154.

[162] J. Chung , P. Khadka , and I. K. Chung , “Nuclear Import of hTERT Requires a Bipartite Nuclear Localization Signal and Akt‐Mediated Phosphorylation,” Journal of Cell Science 125 (2012): 2684–2697. 22366458 10.1242/jcs.099267

[163] M. Liu , Y. Zhang , Y. Jian , et al., “The Regulations of Telomerase Reverse Transcriptase (TERT) in Cancer,” Cell Death & Disease 15 (2024): 90. 38278800 10.1038/s41419-024-06454-7 PMC10817947

[164] S. Ahmed , J. F. Passos , M. J. Birket , et al., “Telomerase Does Not Counteract Telomere Shortening but Protects Mitochondrial Function Under Oxidative Stress,” Journal of Cell Science 121 (2008): 1046–1053. 18334557 10.1242/jcs.019372

[165] D. M. Gordon and J. H. Santos , “The Emerging Role of Telomerase Reverse Transcriptase in Mitochondrial DNA Metabolism,” Journal of Nucleic Acids 2010 (2010): 390791. 20936168 10.4061/2010/390791 PMC2945669

[166] J. Haendeler , S. Dröse , N. Büchner , et al., “Mitochondrial Telomerase Reverse Transcriptase Binds to and Protects Mitochondrial DNA and Function From Damage,” Arteriosclerosis, Thrombosis, and Vascular Biology 29 (2009): 929–935. 19265030 10.1161/ATVBAHA.109.185546

[167] N. K. Sharma , A. Reyes , P. Green , et al., “Human Telomerase Acts as a hTR‐Independent Reverse Transcriptase in Mitochondria,” Nucleic Acids Research 40 (2012): 712–725. 21937513 10.1093/nar/gkr758 PMC3258147

[168] N. Ale‐Agha , P. Jakobs , C. Goy , et al., “Mitochondrial Telomerase Reverse Transcriptase Protects From Myocardial Ischemia/Reperfusion Injury by Improving Complex I Composition and Function,” Circulation 144 (2021): 1876–1890. 34672678 10.1161/CIRCULATIONAHA.120.051923

[169] S. Li , Q. Xin , G. Fang , et al., “Upregulation of Mitochondrial Telomerase Reverse Transcriptase Mediates the Preventive Effect of Physical Exercise on Pathological Cardiac Hypertrophy via Improving Mitochondrial Function and Inhibiting Oxidative Stress,” Biochimica et Biophysica Acta (BBA) – Molecular Basis of Disease 1870 (2024): 166859. 37643691 10.1016/j.bbadis.2023.166859

[170] Y. Zhang , J. He , S. Ling , et al., “Injectable Gene/Fiber‐Plexes Reverse Adipose Niche Senescence via Mito‐Tert Activation by Endogenous Mitochondrial Translocation,” Advanced Functional Materials 35 (2025): 2415080.

[171] A. Melber and C. M. Haynes , “UPR^mt^ Regulation and Output: A Stress Response Mediated by Mitochondrial‐Nuclear Communication,” Cell Research 28 (2018): 281–295. 29424373 10.1038/cr.2018.16 PMC5835775

[172] I. Pimenta de Castro , A. C. Costa , D. Lam , et al., “Genetic Analysis of Mitochondrial Protein Misfolding in *Drosophila melanogaster* ,” Cell Death & Differentiation 19 (2012): 1308–1316. 22301916 10.1038/cdd.2012.5 PMC3392634

[173] E. Owusu‐Ansah , W. Song , and N. Perrimon , “Muscle Mitohormesis Promotes Longevity via Systemic Repression of Insulin Signaling,” Cell 155 (2013): 699–712. 24243023 10.1016/j.cell.2013.09.021 PMC3856681

[174] S. A. Dogan , C. Pujol , P. Maiti , et al., “Tissue‐Specific Loss of DARS2 Activates Stress Responses Independently of Respiratory Chain Deficiency in the Heart,” Cell Metabolism 19 (2014): 458–469. 24606902 10.1016/j.cmet.2014.02.004

[175] M. Song , K. Mihara , Y. Chen , L. Scorrano , and G. W. Dorn II , “Mitochondrial Fission and Fusion Factors Reciprocally Orchestrate Mitophagic Culling in Mouse Hearts and Cultured Fibroblasts,” Cell Metabolism 21 (2015): 273–286. 25600785 10.1016/j.cmet.2014.12.011 PMC4318753

[176] N. A. Khan , M. Auranen , I. Paetau , et al., “Effective Treatment of Mitochondrial Myopathy by Nicotinamide Riboside, a Vitamin B3,” EMBO Molecular Medicine 6 (2014): 721–731. 24711540 10.1002/emmm.201403943 PMC4203351

[177] M. Mohrin , J. Shin , Y. Liu , et al., “A Mitochondrial UPR‐Mediated Metabolic Checkpoint Regulates Hematopoietic Stem Cell Aging,” Science 347 (2015): 1374–1377. 25792330 10.1126/science.aaa2361 PMC4447312

[178] J. Durieux , S. Wolff , and A. Dillin , “The Cell‐Non‐Autonomous Nature of Electron Transport Chain‐Mediated Longevity,” Cell 144 (2011): 79–91. 21215371 10.1016/j.cell.2010.12.016 PMC3062502

[179] J. M. Copeland , J. Cho , T. Lo Jr. , et al., “Extension of Drosophila Life Span by RNAi of the Mitochondrial Respiratory Chain,” Current Biology 19 (2009): 1591–1598. 19747824 10.1016/j.cub.2009.08.016

[180] C. Lee , J. Zeng , B. G. Drew , et al., “The Mitochondrial‐Derived Peptide MOTS‐c Promotes Metabolic Homeostasis and Reduces Obesity and Insulin Resistance,” Cell Metabolism 21 (2015): 443–454. 25738459 10.1016/j.cmet.2015.02.009 PMC4350682

[181] C. Lee , K. Yen , and P. Cohen , “Humanin: A Harbinger of Mitochondrial‐Derived Peptides?,” Trends in Endocrinology & Metabolism 24 (2013): 222–228. 23402768 10.1016/j.tem.2013.01.005 PMC3641182

[182] K. H. Kim , Y. T. Jeong , H. Oh , et al., “Autophagy Deficiency Leads to Protection From Obesity and Insulin Resistance by Inducing Fgf21 as a Mitokine,” Nature Medicine 19 (2013): 83–92. 10.1038/nm.3014 23202295

[183] H. Bao , J. Cao , M. Chen , et al., “Biomarkers of Aging,” Science China Life Sciences 66 (2023): 893–1066. 37076725 10.1007/s11427-023-2305-0 PMC10115486

[184] T. Shpilka and C. M. Haynes , “The Mitochondrial UPR: Mechanisms, Physiological Functions and Implications in Ageing,” Nature Reviews Molecular Cell Biology 19 (2018): 109–120. 29165426 10.1038/nrm.2017.110

[185] H. Chen , X. Chen , Z. Zhou , et al., “Mesenchymal Stromal Cell‐Mediated Mitochondrial Transfer Unveils New Frontiers in Disease Therapy,” Stem Cell Research & Therapy 16 (2025): 546. 41063290 10.1186/s13287-025-04675-x PMC12505854

[186] H. Liu , H. Mao , X. Ouyang , R. Lu , and L. Li , “Intercellular Mitochondrial Transfer: The Novel Therapeutic Mechanism for Diseases,” Traffic 25 (2024): e12951. 39238078 10.1111/tra.12951

[187] X. Miao , P. Jiang , Z. Wang , W. Kong , and L. Feng , “Mitochondrial Transplantation: A Novel Therapeutic Approach for Treating Diseases,” MedComm 6 (2025): e70253. 40502813 10.1002/mco2.70253 PMC12152381

[188] Y. Gao , L. Guo , F. Wang , Y. Wang , P. Li , and D. Zhang , “Development of Mitochondrial Gene‐Editing Strategies and Their Potential Applications in Mitochondrial Hereditary Diseases: A Review,” Cytotherapy 26 (2024): 11–24. 37930294 10.1016/j.jcyt.2023.10.004

[189] P. Shelke , S. Tribhuvan , A. K. Agrahari , and R. Saxena , “The Evolving Landscape of Mitochondrial Base Editing: Advances in Precision, Modeling, and Therapeutic Potential,” Mitochondrion 86 (2026): 102093. 41173131 10.1016/j.mito.2025.102093

[190] N. Li , B. Wu , Y. Xiao , et al., “Tools and Delivery Technologies for Mitochondrial Gene Editing,” Cell Biomaterials 2 (2026): 100254.

[191] Y. Jang and K. Lim , “Recent Advances in Mitochondria‐Targeted Gene Delivery,” Molecules 23 (2018): 2316. 30208599 10.3390/molecules23092316 PMC6225103

[192] H. Yu , R. D. Koilkonda , T. H. Chou , et al., “Gene Delivery to Mitochondria by Targeting Modified Adenoassociated Virus Suppresses Leber's Hereditary Optic Neuropathy in a Mouse Model,” Proceedings of the National Academy of Sciences of the United States of America 109 (2012): E1238–E1247. 22523243 10.1073/pnas.1119577109 PMC3356643

[193] Y. Li , X. Li , L. Wei , and J. Ye , “Advancements in Mitochondrial‐Targeted Nanotherapeutics: Overcoming Biological Obstacles and Optimizing Drug Delivery,” Frontiers in Immunology 15 (2024): 1451989. 39483479 10.3389/fimmu.2024.1451989 PMC11524880

[194] Y. Lin , X. Chen , K. Wang , L. Liang , and H. Zhang , “An Overview of Nanoparticle‐Based Delivery Platforms for mRNA Vaccines for Treating Cancer,” Vaccines 12 (2024): 727. 39066365 10.3390/vaccines12070727 PMC11281455

[195] Y. H. Lee , J. Y. Park , H. Lee , et al., “Targeting Mitochondrial Metabolism as a Strategy to Treat Senescence,” Cells 10 (2021): 3003. 34831224 10.3390/cells10113003 PMC8616445

[196] X. Zhou , S. Liu , Y. Lu , M. Wan , J. Cheng , and J. Liu , “MitoEVs: A New Player in Multiple Disease Pathology and Treatment,” Journal of Extracellular Vesicles 12 (2023): e12320. 37002588 10.1002/jev2.12320 PMC10065981

[197] J. Novak , Z. Nahacka , G. L. Oliveira , et al., “The Adaptor Protein Miro1 Modulates Horizontal Transfer of Mitochondria in Mouse Melanoma Models,” Cell Reports 44 (2025): 115154. 39792553 10.1016/j.celrep.2024.115154

[198] P. Lou , X. Zhou , Y. Zhang , et al., “Harnessing Tissue‐Derived Mitochondria‐Rich Extracellular Vesicles (Ti‐MitoEVs) to Boost Mitochondrial Biogenesis for Regenerative Medicine,” Science Advances 11 (2025): eadt1318. 40668934 10.1126/sciadv.adt1318 PMC12266123

